# Large-Scale Single-Cell and Bulk Sequencing Analyses Reveal the Prognostic Value and Immune Aspects of CD147 in Pan-Cancer

**DOI:** 10.3389/fimmu.2022.810471

**Published:** 2022-04-06

**Authors:** Jingwei Zhang, Zeyu Wang, Xun Zhang, Ziyu Dai, Wen Zhi-Peng, Jing Yu, Yun Peng, Wantao Wu, Nan Zhang, Peng Luo, Jian Zhang, Zaoqu Liu, Songshan Feng, Hao Zhang, Quan Cheng

**Affiliations:** ^1^ Department of Neurosurgery, Xiangya Hospital, Central South University, Changsha, China; ^2^ National Clinical Research Center for Geriatric Disorders, Xiangya Hospital, Central South University, Changsha, China; ^3^ Department of Pharmacy, The Affiliated Hospital of Guizhou Medical University, Guizhou Medical University, Guiyang, China; ^4^ Department of Clinical Pharmacology, Xiangya Hospital, Central South University, Changsha, China; ^5^ Department of Geriatrics, Xiangya Hospital, Central South University, Changsha, China; ^6^ Teaching and Research Section of Clinical Nursing, Xiangya Hospital of Central South University, Changsha, China; ^7^ Department of Oncology, Xiangya Hospital, Central South University, Changsha, China; ^8^ One-third Lab, College of Bioinformatics Science and Technology, Harbin Medical University, Harbin, China; ^9^ Department of Oncology, Zhujiang Hospital, Southern Medical University, Guangzhou, China; ^10^ Department of Interventional Radiology, The First Affiliated Hospital of Zhengzhou, Zhengzhou, China; ^11^ Xiangya Cancer Center, Xiangya Hospital, Central South University, Changsha, China; ^12^ Key Laboratory of Molecular Radiation Oncology Hunan Province, Changsha, China; ^13^ Clinical Diagnosis and Therapy Center for Glioma of Xiangya Hospital, Central South University, Changsha, China

**Keywords:** CD147, pan-cancer, immunotherapy, macrophages, tumor microenvironment

## Abstract

CD147 plays an important role in promoting tumor proliferation and inhibiting cancer cell apoptosis in the tumor microenvironment. However, the mechanisms by which CD147 is involved in tumorigenesis remains unclear. This study systematically analyzed the prognostic value and immune characteristics of CD147 in 31 cancer types. The expression levels and mutant landscapes of CD147 in pan-cancer were explored. The Kaplan-Meier (KM) analysis was applied to analyze the prognostic value of CD147. The immune characteristics of CD147 in the tumor microenvironment were evaluated *via* TIMER 2.0 and R package (immunedeconv). We also explored the expression of CD147 on tumor cells and stromal cells through Gene Set Variation Analysis and single-cell sequencing analysis. The co-expression of CD147 and macrophage markers CD68 and CD163 in pan-cancer was detected using multiplex immunofluorescence staining on tissue microarrays. CD147 was found to be overexpressed in almost all cancer types, which was related to poor outcome. CD147 expression exhibited a strong association with immune infiltrates, immune checkpoint molecules, and neoantigen levels in the tumor microenvironment. In addition, CD147 was expressed on various cell types in the tumor microenvironment, including tumor cells, macrophages, T cells, monocytes, fibroblasts, etc. Furthermore, multiplex immunofluorescence revealed the co-expression pattern of CD147 and macrophage markers CD68 and CD163 in many tumor types. Finally, the immunotherapy response and sensitive small molecule drugs based on CD147 expression were predicted. In sum, CD147 has a significant relationship with the clinical outcome and immune infiltrates in multiple cancer types. Inhibiting the CD147-dependent signaling pathways might be a promising therapeutic strategy for tumor immunotherapy.

## Introduction

Worldwide, cancer is listed as the main cause of death and has become a major obstacle to increasing life expectancy in almost every country (1, 2). More than 19 million new cancer cases and nearly 10 million cancer deaths occurred in the past year based on the global cancer epidemiological survey estimated by the International Agency for Research on Cancer (3). By 2040, an estimated 30 million newly diagnosed cases and almost 17 million cancer-related deaths are expected to occur per year (2, 4). In general, countries with the highest population life expectancy, education, and living standards have the highest incidence of cancer (5). After many years of disappointing therapeutic results with traditional strategies, immunotherapy has become a promising tool for cancer treatment. In particular, immune checkpoint-based immunotherapy shows remarkable clinical benefits in prolonging the survival time of cancer patients (6). Tumor microenvironment (TME) is a complex and dynamic environment around tumor consisting of surrounding immune cells, signaling molecules, blood vessels, and the extracellular matrix (ECM) (7, 8). Immune checkpoints maintain an intimate relationship with immune cells in TME, such as regulatory T cells (Tregs), macrophages, natural killer (NK) cells, astrocytes, B cells, etc. Programmed death 1 (PD-1)/programmed cell death-ligand 1 (PD-L1) and cytotoxic T-lymphocyte-associated antigen 4 (CTLA-4)/B7 are the two most important immune checkpoint signaling pathways (9). PD-1 and CTLA-4 are immune inhibitory receptors expressed by activated T cells, which negatively regulate T cell immune function during different phases of T-cell activation in TME (10, 11). Specific inhibitors that target PD-1/PD-L1 and CTLA-4 have undergone a large number of clinical trials and been approved by the FDA, and are widely used in many cancer types. Thus, it is necessary to explore novel and effective immunosuppressive points in tumor immunotherapy.

CD147 (cluster of differentiation 147) is an important cell-surface glycoprotein that is expressed on various cell types, such as tumor cells, epithelial cells, cancer-associated fibroblasts (CAFs), T cells, peripheral monocytes, etc. (12, 13). CD147 was first identified as a modulator of matrix metalloproteinases (MMPs) and then found to play a vital role in cancer therapy (14). Increasing studies indicated that CD147 promotes tumor progression through several mechanisms, including reprogramming glycolytic metabolism, inducing matrix degradation, promoting tumor cell invasion and metastasis (15). Studies found that CD147 was overexpressed in the triple-negative breast cancer (TNBC) tissues and positively related to progression, TNM stage, and lymph node metastasis (16). Down-regulating the expression of CD147 by MiR-890 could significantly promote TNBC cell apoptosis through caspase-3 signaling (17). Moreover, strategies that inhibited CD147 expression *via* lentivirus carrying CD147 shRNA or cDNA showed potential therapeutic effectiveness for lethal metastatic breast cancer through eliminating activated cancer stem cells (CSCs) (18). However, the specific mechanisms through which CD147 involved in the pan-cancer immunity and progression remain ill-defined. Moreover, the relationship between CD147 levels and immune infiltrates in the TME has not been fully studied.

Therefore, in this study, we explored the prognostic value of CD147 in 31 cancer types using large-scale RNA-sequencing (RNA-seq) data from the TCGA. Meanwhile, we explored the role of CD147 in immune infiltration in the TME using online databases. In addition, the expression landscapes of CD147 on tumor and stromal cells through Gene Set Variation Analysis, single-cell sequencing level, and tumor tissue level. Moreover, we predicted the immunotherapy response and sensitive small molecule drugs based on CD147 expression from the public databases.

## Materials and Methods

### Datasets Collecting and Preprocessing

The RNA-seq data of pan-cancer samples including adrenocortical carcinoma (ACC), bladder cancer (BLCA), breast carcinoma (BRCA), cervical cancer (CESC), cholangiocarcinoma (CHOL), colorectal cancer (COAD), esophageal cancer (ESCA), glioblastoma (GBM), head and neck squamous cell carcinoma (HNSC), kidney chromophobe (KICH), kidney renal clear cell carcinoma (KIRC), kidney renal papillary cell carcinoma (KIRP), low grade glioma (LGG), liver hepatocellular carcinoma (LIHC), lung adenocarcinoma (LUAD), lung squamous cell carcinoma (LUSC), mesothelioma (MESO), ovarian serous cystadenocarcinoma (OV), pancreatic adenocarcinoma (PAAD), pheochromocytoma and paraganglioma (PCPG), prostate cancer (PRAD), rectal cancer (READ), sarcoma (SARC), skin cutaneous melanoma (SKCM), stomach adenocarcinoma (STAD), testicular cancer (TGCT), thyroid carcinoma (THCA), thymoma (THYM), uterine corpus endometrial carcinoma (UCEC), uterine carcinosarcoma (UCS) and ocular melanomas (UVM), were obtained from The Cancer Genome Atlas (TCGA) dataset (https://portal.gdc.cancer.gov/), which samples with complete survival information of OS and DSS and tumor histology were included. The normal samples were selected from the Genotype-Tissue Expression (GETx) dataset. The combined cohort of TCGA and GTEx samples was downloaded from https://xenabrowser.net/, which batch effect was already removed (CutAdapt was used for adapter trimming, STAR was used for alignment, and RSEM and Kallisto were used as quantifiers). The cell lines data were downloaded from the Cancer Cell Line Encyclopedia (CCLE) and Human Protein Atlas (HPA) datasets. The single-cell sequencing datasets of invasive breast carcinoma (BRCA, GSE75688, and GSE118389), cholangiocarcinoma (CHOL, GSE125449), colon adenocarcinoma (COAD, GSE81861), head and neck squamous cell carcinoma (HNSC, GSE103322), liver hepatocellular carcinoma (LIHC, GSE125449), bladder cancer (BLCA, GSE145137), kidney renal clear cell carcinoma (KIRC, GSE121636 and GSE171306), ovarian serous cystadenocarcinoma(OV, GSE118828), prostate adenocarcinoma (PRAD, GSE137829), skin cutaneous melanoma (SKCM, GSE72056), stomach adenocarcinoma (STAD, GSE183904) were downloaded from the GEO database (https://www.ncbi.nlm.nih.gov/geo/); the single-cell sequencing dataset of glioblastoma multiforme (GBM, SCP50, and SCP393) were downloaded from the Single Cell Portal platform (http://singlecell.broadinstitute.org); the single-cell sequencing dataset of LUAD was downloaded from the BioProject (PRJNA591860).

### Identification of Corresponding Characteristics

The CBIOPORTAL database (https://www.cbioportal.org/) was used to analyze the mutant genetic aspects of CD147. The disease-specific survival (DSS) and overall survival (OS) were obtained from the KM analysis. The immune landscape (immune related characteristics) of CD147 were analyzed by the ESTIMATE (Estimation of stromal and Immune cells in malignant Tumor tissues using Expression) and immunedeconv (TIMER, xCell, MCP-counter, CIBERSORT, EPIC, and quanTIseq) algorithms. ESTIMATE algorithm was reported to assess the infiltration level of stromal cells and immune cells in tumor tissue. The ESTIMATE algorithm consists of three score systems as follows: stromal score (positively correlating with the presence of stroma), immune score (positively correlating with the level of immune cells infiltrations) and estimate score (which infers and negatively correlates with tumor purity). The biological functions and pathways of CD147 were analyzed by the gene set variation analysis (GSVA) for gene ontology (GO) terms and the gene set enrichment analysis (GSEA) for Kyoto Encyclopedia of Genes and Genomes database (KEGG) terms and HALLMARK terms. The immunotherapy and gene treatment responses of CD147 were predicted from the TIDE (http://tide.dfci.harvard.edu) and TISMO (http://tismo.cistrome.org) websites. The protein-protein interaction network (PPI) analysis was conducted *via* STRING (https://string-db.org/cgi/input.pl) website. The involvement of CD147 in diseases and aid systematic drug target identification and prioritization was identified from the OPEN TARGET platform (https://www.targetvalidation.org/).

### Single-Cell Sequencing Analysis

We integrated the BRCA data through the Anchors function from the R package Seurat. R package Seurat was used for quality control (19). Principal component analysis (PCA) was performed for dimension reduction. Cells were clustered together using the FindClusters function. The R package infercnv and copykat were used for the identification of tumor cells. The UMAP function was used for dimensionality reduction of visualization. Vlnplot, Dimplot, and Featureplot were used for visualizing CD147 levels.

### Multiplex Immunofluorescence Staining

Multiplex immunofluorescence staining was performed as previously described (20, 21). The primary Abs were CD147 (Rabbit, 1:100, Proteintech, China), CD68 (Rabbit, 1:3000, AiFang biological, China), CD163 (Rabbit, 1:3000, Proteintech, China). The secondary antibody was horseradish peroxidase-conjugated secondary antibody incubation (GB23301, GB23303, Servicebio, China), and the tyramide signal amplification was TSA (FITC-TSA, CY3-TSA, 594-TSA, and 647-TSA (Servicebio, China)). Multispectral images were analyzed, and positive cells were quantified at a single-cell level by Caseviewer (C.V 2.3, C.V 2.0) and Pannoramic viewer (P.V 1.15.3) image analysis software. Negative control procedures included the omission of the primary antibody.

### Statistical Analysis

The student’s t-test and the Kruskal-Wallis test were used to compare CD147 levels in the tumor and normal tissues. The cutoff value of CD147 was calculated by the R package survminer. Samples were grouped into CD147-high and CD147-low groups based on the cutoff value. The survival differences between CD147-high and CD147-low groups regarding OS and DSS were explored by the log-rank test. All tests were two-sided, and P < 0.05 was considered to be statistically significant.

## Results

### Expression and Mutant Aspects of CD147

**Figure 1:**
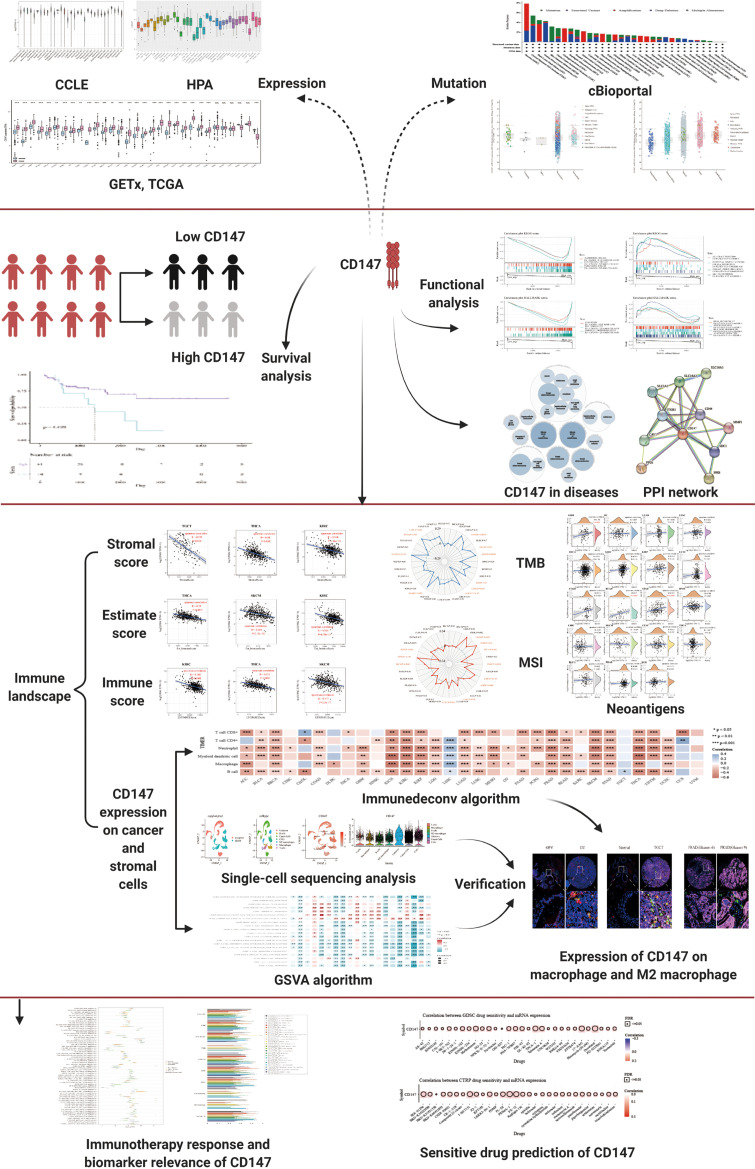
The flow chart of the entire study.

**Figure 2:**
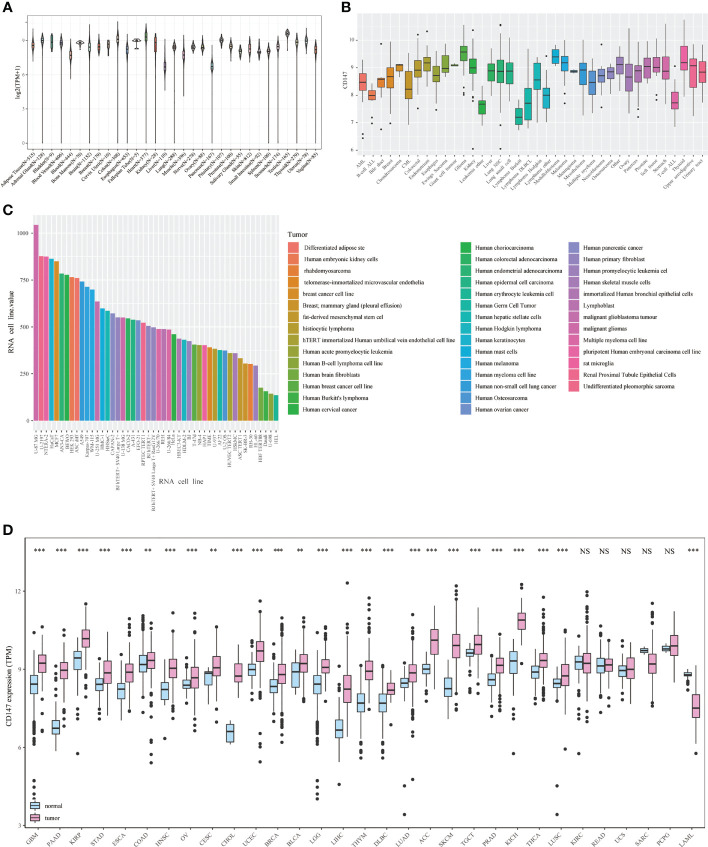
Expression landscape of CD147 in the normal and tumor samples. CD147 levels in 31 human tissues from the GETx dataset **(A)**. CD147 levels in tumor cell lines from the CCLE dataset **(B)**. CD147 levels in 43 tumor cell lines from the HPA dataset **(C)**. CD147 expression analyzed by TCGA and GETx datasets **(D)**. **p < 0.01, ***p < 0.001, NS, no significant differences.

In this paper, pan-cancer samples of TCGA database were included for subsequent analysis. The flow chart of our study design is shown in **Figure 1**. The sample ids and dataset ids in this paper is listed in **Supplementary Table 1**. To fully elaborate the expression of CD147 in the normal and cancer samples. First, we first observed the expression levels of CD147 in the 31 normal tissues based on the GTEx dataset (**Figure 2A**). The top three CD147-enriched tissues were testis, heart, and colon. Data from the CCLE dataset indicated that CD147 was highly expressed in these 38 tumor cell lines, especially in glioma, medulloblastoma, melanoma, endometrium, and thyroid (**Figure 2B**). Data from the HPA dataset indicated that the top five CD147 RNA-enriched cell lines were U-87, U-2197, NTERA-2, HaCaT, and MCF7 (**Figure 2C**). Data from the TCGA dataset indicated that CD147 upregulated in acute myeloid leukemia tissue than normal control (**Figure 2D**; P<0.001). In particular, we found that CD147 was significantly increased in cancer samples than normal controls in almost all cancers except KIRC, LAML, READ, UCS, SARC, and pheochromocytoma and paraganglioma (PCPG) (**Figure 2D**; P<0.01).

In addition, we explored the mutant aspects of CD147 in pan-cancer using the cBioportal dataset (**Supplementary Figure 1**). The results demonstrated that SARC, CESC, LGG, and OV had a high mutation level, with an alteration frequency of more than 4% (**Supplementary Figures 1A, B**). We also analyzed the relationship between CD147 mRNA levels and mutant types (**Supplementary Figure 1C**) and copy-number alterations (**Supplementary Figure 1D**). A total of 64 mutation sites (including 52 missenses, eight truncating, three splices, and one fusion) were found between amino acids 0 and 385 (**Supplementary Figure 1E**).

### Prognostic Value of CD147

**Figure 3:**
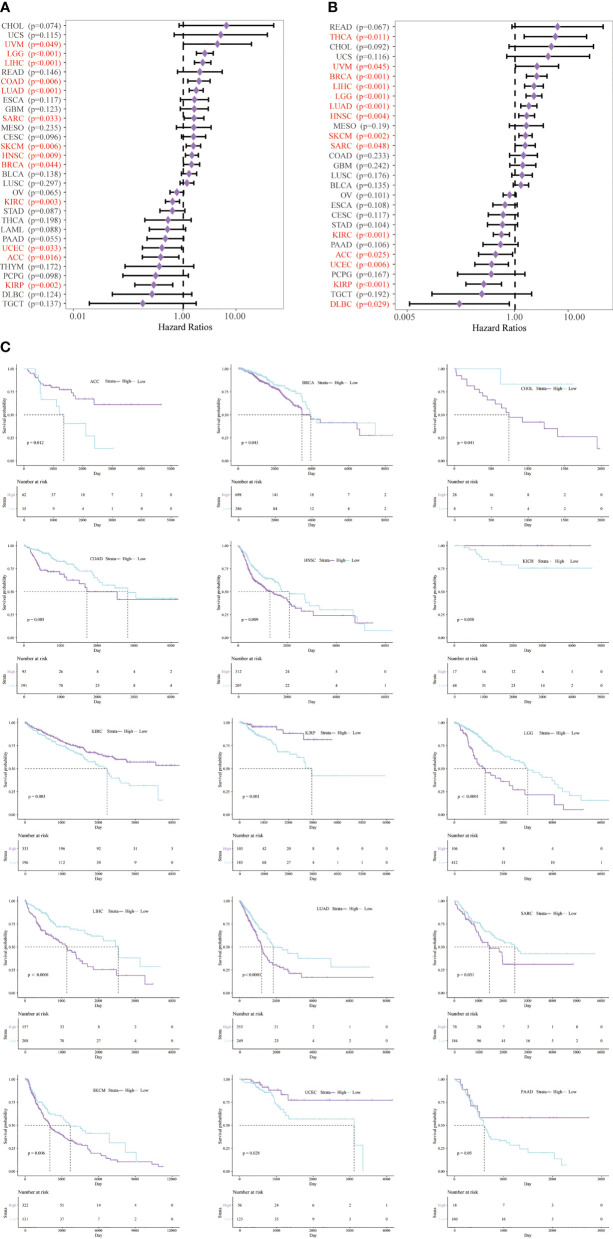
Survival analysis of CD147 in pan-cancer from the TCGA database. Survival analysis of CD147 on OS **(A)** and DSS **(B)** in pan-cancer described by the forest plot. The prognostic value of CD147 on OS displayed by the KM method **(C)**.

Next, we studied the prognostic value of CD147 in pan-cancer through KM analysis. CD147 exhibited remarkable value in predicting OS (**Figure 3A**) and DSS (**Figure 3B**) in many tumor types. Elevated CD147 was related to shorter OS in BRCA, CHOL, COAD, HNSC, LGG, LIHC, LUAD, SARC, and SKCM (**Figure 3C**; P<0.05). On the contrary, increased CD147 was associated with longer OS in ACC, KICH, KIRC, KIRP, UCEC (**Figure 3C**; P<0.05). In addition, overexpressed CD147 was related to decreased DSS in BRCA, HNSC, LGG, LIHC, LUAD, READ, SARC, SKCM, and THCA while related to prolonged DSS in ACC, lymphoid neoplasm diffuse large B-cell lymphoma (DLBC), KIRC, KIRP, UCEC (**Supplementary Figure 2**; P<0.05).

### Immune Aspects of CD147 in the Tumor Microenvironment

To identify the immune aspects of CD147 in the TME in pan-cancer, we calculated the correlation between CD147 levels and the immune scores (**Supplementary Figure 3**), estimate scores (**Supplementary Figure 4**), and stromal scores (**Supplementary Figure 5**) in 33 cancer types based on the ESTIMATE algorithm. The top three tumors with the significant correlation between CD147 and stromal scores were TGCT, THCA, and KIRC; the top three tumors with the significant correlation between CD147 and immune scores were THCA, SKCM, and KIRC; the top three tumors with the significant correlation between CD147 and estimating scores were KIRC, THCA, and SKCM (**Supplementary Figure 6A**; P<0.0001). BRCA, KIRC, and LIHC were the top three cancers with the significant correlation between CD147 and the immune infiltrates in the TME, such as B cell, CD4 and CD8 T cell, dendritic cell, macrophage, and neutrophil (**Supplementary Figure 6B**; P<0.01). We also studied the correlation between CD147 and neoantigens in human cancers (**Supplementary Figure 7**). Our data indicated that upregulated CD147 levels were significantly related to the number of neoantigens in GBM, READ, and STAD (p<0.001).

**Figure 4:**
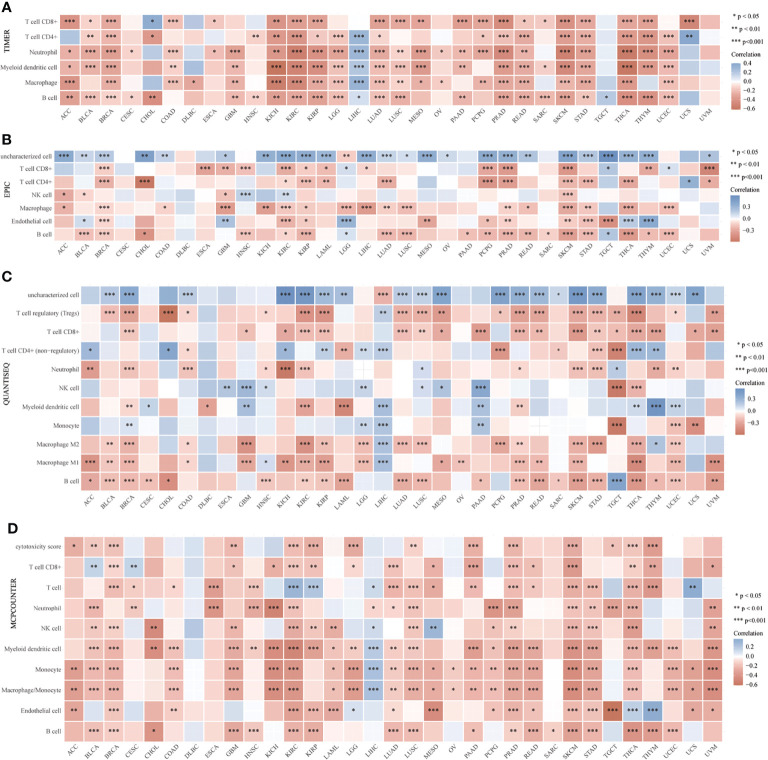
Relationship between CD147 levels and immune infiltrates analyzed by the R package immunedeconv in the TME. Immune cell infiltration analyzed by the TIMER **(A)**, EPIC **(B)**, quanTIseq **(C)**, MCP-counter **(D)**, CIBERSORT algorithms. *p < 0.05, **p < 0.01, ***p < 0.001.

In addition, to clarify the relationship between CD147 and immune cells in the TME in pan-cancer, we applied six algorithms for quantification of immune cells, including TIMER (**Figure 4A**), EPIC (**Figure 4B**), quanTIseq (**Figure 4C**), MCP-counter (**Figure 4D**), CIBERSORT (**Supplementary Figure 8A**), and xCell (**Supplementary Figure 8B**). Results indicated that CD147 kept an intimate relationship with these immune cells in pan-cancer. Especially, elevated CD147 maintained a significant positive correlation with CD4^+^ T cell, CD8^+^ T cell, neutrophils, B cells, and macrophages in BRCA, KIRC, KIRP, PRAD, and SKCM (**Figures 4A, B, D**; P<0.05). Increased CD147 showed a significant correlation with M1 or M2 macrophages in BLCA, BRCA, COAD, GBM, KIRC, PRAD, LIHC, PRAD, LUAD, STAD, and SKCM (**Figure 4C** and **Supplementary Figure 8**; P<0.05).

**Figure 5:**
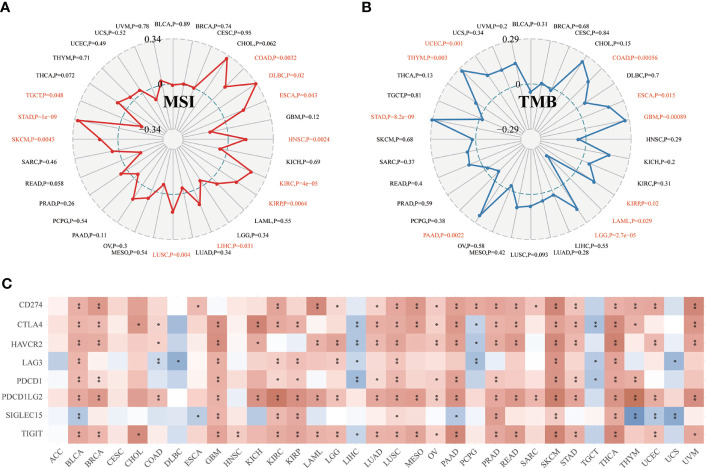
Relationship between CD147 expression and MSI, TMB, and immune checkpoints in pan-cancer. Relationship between CD147 expression and MSI displayed by the radar chart **(A)**. Relationship between CD147 expression and TMB displayed by the radar chart **(B)**. Relationship between CD147 expression and immune checkpoints **(C)**. *p< 0.05, **p < 0.01.

Moreover, we found that CD147 positively related to microsatellite instability (MSI) in COAD, DLBC, ESCA, HNSC, KIRC, KIRP, LIHC, LUSC, SKCM, and STAD (P<0.05), while negatively related to MSI in TGCT (**Figure 5A**; P<0.05). CD147 was positively related to tumor mutation burden (TMB) in COAD, ESCA, GBM, KIRP, LGG, PAAD, STAD, THYM, and UCEC (P<0.05), while negatively associated with TMB in LAML (**Figures 5B**; P<0.05). We then explored the relationship between CD147 expression and classic immune checkpoints, such as SIGLEC15, TIGIT, CD274, HAVCR2, PDCD1, CTLA4, LAG3, and PDCD1LG2 (**Figure 5C**), which indicated that most of these immune checkpoints had a close correlation with CD147 expression levels, especially in BLCA, GBM, KIRC, PAAD, PRAD, LUSC, SKCM, and THCA. In summary, CD147 plays a vital role in immune infiltrates in pan-cancer and might act as a novel immunotherapy target in tumor therapy.

### Functional Analysis Based on CD147 Expression

**Figure 6:**
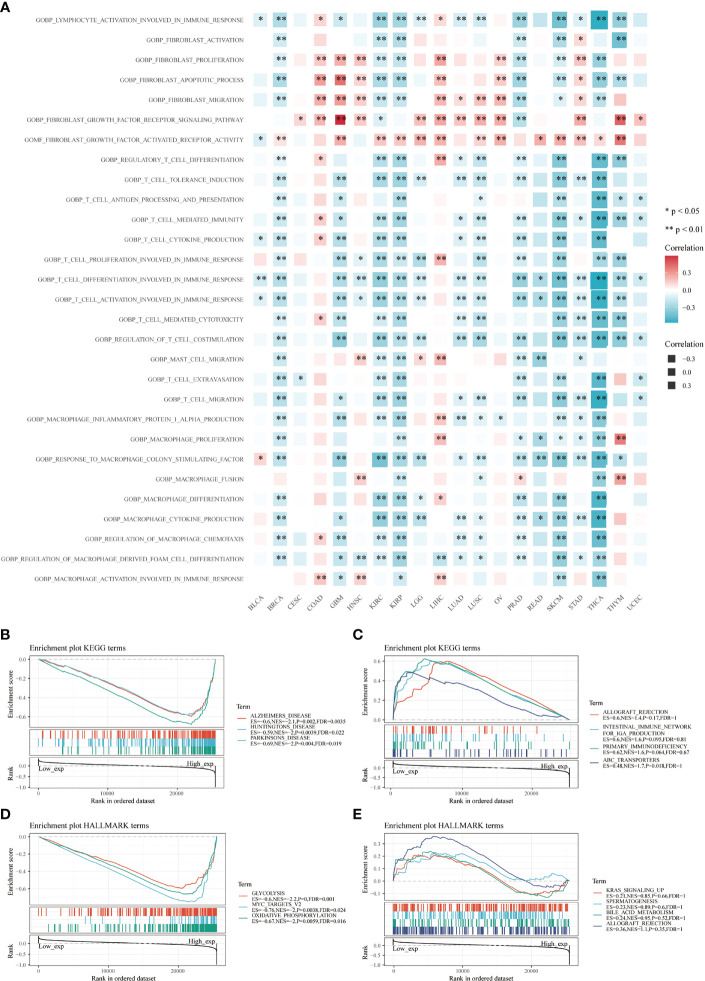
Functional analysis based on CD147 levels in pan-cancer. Functional enrichment pathways of CD147 based on the GSVA algorithm **(A)**. Top three negative **(B)** and top four positive **(C)** enriched pathways based on the KEGG terms. Top three negative **(D)** and top four positive **(E)** enriched pathways based on the HALLMARK terms.

CD147 was negatively related to immune-related pathways in various cancers based on GO terms calculated by GSVA, especially in BRCA, GBM, KIRC, KIRP, PRAD, SKCM, and THCA (**Figure 6A**; P<0.05). Interestingly, most of these pathways were involved in the activation and proliferation of fibroblasts, T cells, and macrophages related pathways. Meanwhile, we found that the top three negatively enriched pathways were Alzhemers disease, Huntingtons disease, Parkinsons disease, glutathione metabolism, and pyruvate metabolism (**Figure 6B**; P<0.01), while the top four positively enriched pathways were ABC transporters based on the KEGG terms (**Figure 6C**; P<0.05). Glycolysis, MYC targets, and oxidative phosphorylation were the top three negatively enriched pathways based on the HALLMARK terms (**Figure 6D**; P<0.01), while the top four positively enriched pathways were KRAS signaling up, spermatogenesis, bile acid metabolism, and allograft rejection based on the HALLMARK terms (**Figure 6E**; P>0.05).

### Single Cell Sequencing and Multiplex Immunofluorescence Staining of CD147 Expression on Tumor and Stromal Cells

**Figure 7:**
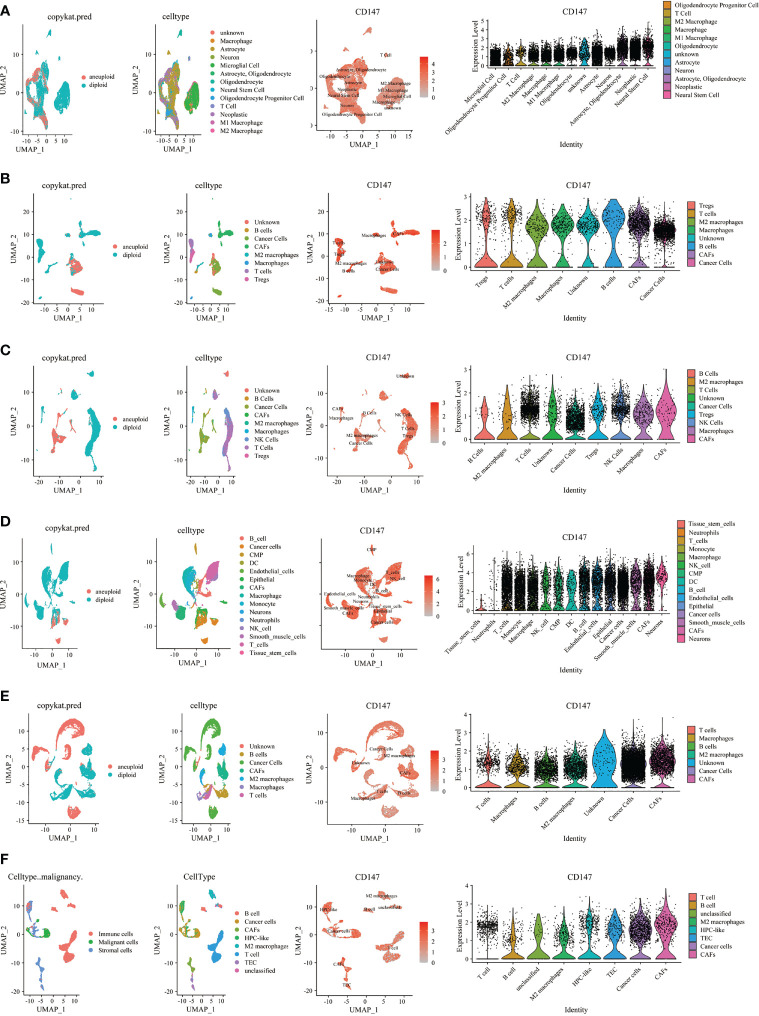
Single cell sequencing analyzing CD147 co-expression on tumor and stromal cells in pan-cancer. The expression levels of CD147 in tumor and stromal cells in GBM **(A)**, HNSC **(B)**, KIRC **(C)**, LUAD **(D)**, PRAD **(E)**, CHOL **(F)**.

Then, we investigated CD147 expression on tumor and stromal cells in several cancer types, including GBM (**Figure 7A**), HNSC (**Figure 7B**), KIRC (**Figure 7C**), LUAD (**Figure 7D**), PRAD (**Figure 7E**), CHOL (**Figure 7F**), STAD (**Supplementary Figure 9A**), LIHC **Supplementary Figure 9B**), OV (**Supplementary Figure 9C**), SKCM (**Supplementary Figure 9D**), COAD (**Supplementary Figure 9E**), BLCA (**Supplementary Figure 9F**), and BRCA (**Supplementary Figure 9G**). Interestingly, results indicated that CD147 is highly co-expressed on cancer cells and stromal cells in these cancers, especially on M2 macrophages, macrophages, T cells, B cells, and CAFs. We also identified the involvement of CD147 in diseases based on the OPEN TARGET platform (**Supplementary Figure 10A**). The PPI network of CD147 with other molecules was shown in **Supplementary Figure 10B**.

**Figure 8:**
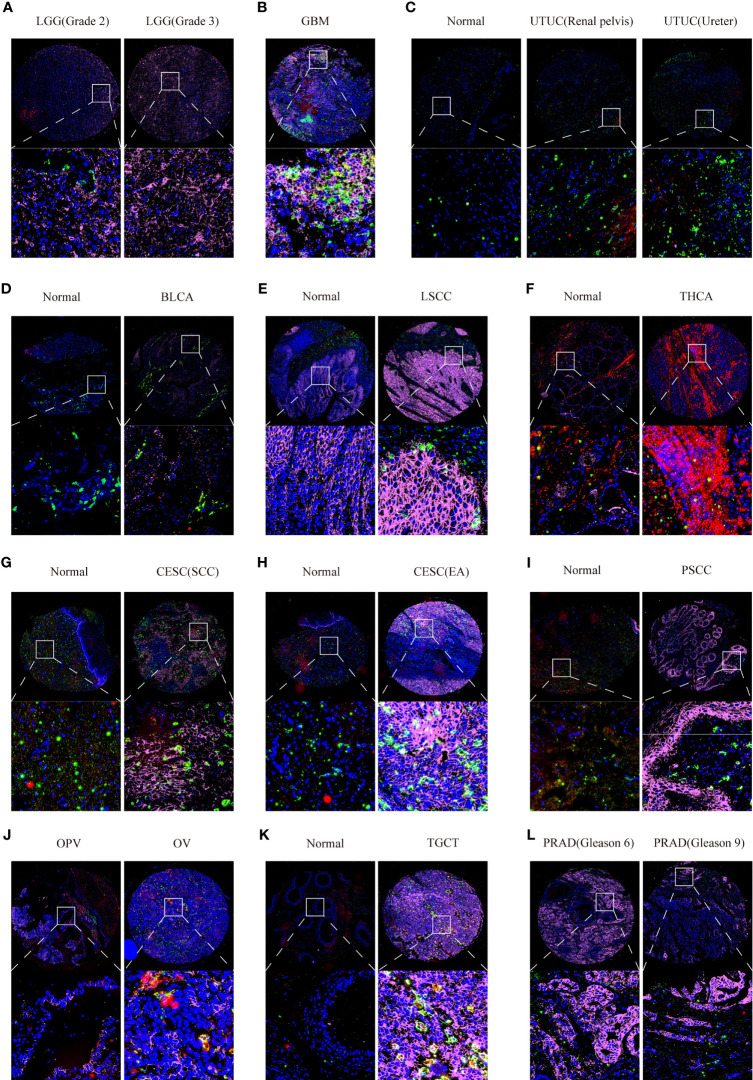
Multiplex immunofluorescence staining analyzing CD147 co-expression on macrophage and M2 macrophage in pan-cancer. The expression levels of CD147 on macrophage and M2 macrophage in LGG **(A)**, GBM **(B)**, UTUC **(C)**, BLCA **(D)**, LSCC **(E)**, THCA **(F)**, CESC **(G, H)**, PSCC **(I)**, OV **(J)**, TGCT **(K)**, and PRAD **(L)**.

Furthermore, we also used multiplex immunofluorescence staining to verify the co-expression of CD147 with macrophages (CD68) and M2 macrophages (CD163) in these cancers, which CD68 was marked red, CD163 was marked green, CD147 was marked purple, and DAPI was marked blue (**Supplementary Figure 10C**). Results indicated that CD147 increased in WHO III gliomas than WHO II gliomas (**Figure 8A**). Data from the multiplex immunofluorescence staining of GBM tissues showed that CD147 was highly expressed on M2 macrophages (**Figure 8B**). We found a lot of CD163 positive cells in UTUC cancer and normal samples (**Figure 8C**). In addition, we found that CD147 elevated in the cancer samples than normal samples in BLCA (**Figure 8D**), laryngeal squamous cell carcinoma (LSCC) (**Figure 8E**), THCA (**Figure 8F**), CESC (**Figures 8G, H**), penile squamous cell carcinoma PSCC (**Figure 8I**), and TGCT (**Figure 8K**). PRAD samples with higher Gleason scores had less CD147 levels than lower Gleason scores (**Figure 8L**). Interestingly, CD147 was found to mainly express on CD163^+^ M2 macrophages in BLCA, LSCC, CESC, PSCC, TGCT, and PRAD. In addition, CD147 was also found co-expressed on CD68^+^ macrophages in UTUC, THCA, BLCA, CESC, Ovarian serous papillary cystadenocarcinoma (OPV) and OV (**Figure 8J**).

### Immunotherapy Response and Sensitive Drugs Prediction Based on CD147 Expression

**Figure 9:**
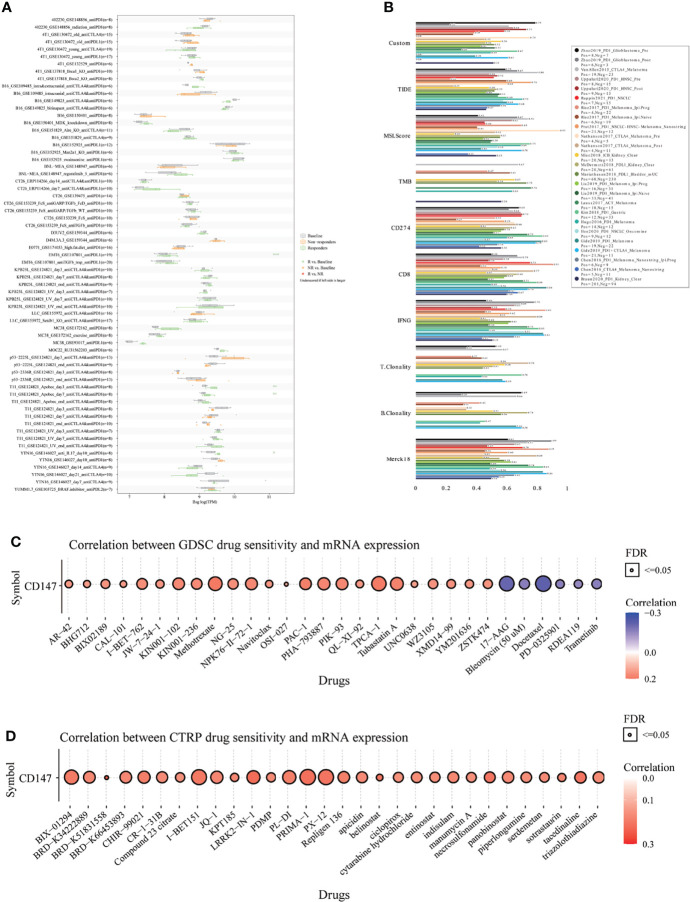
Immunotherapy response, biomarker relevance, and sensitive drug prediction of CD147 in pan-cancer. Immunotherapy response **(A)** and biomarker relevance **(B)** of CD147 in immunotherapy cohorts. Predictive drugs based on the CD147 expression in pan-cancer from the GDSC **(C)** and CTRP **(D)** datasets.

Finally, to fully explore the promising value of CD147 as a novel immune target in pan-cancer, we predicted the immunotherapy response and sensitive drugs based on CD147 expression (**Figure 9**). Notably, CD147 could significantly predict immunotherapy response in 4 murine immunotherapy cohorts, which responders were more likely to have elevated CD147 levels (**Figure 9A**). We also calculated the biomarker relevance of CD147 by comparing it with standardized biomarkers based on their predictive power of immunotherapy response of human immunotherapy cohorts. Interestingly, we found that CD147 alone had an AUC of more than 0.5 in 12 of the 25 immunotherapy cohorts (**Figure 9B**). CD147 exhibited a higher predictive value than TMB, T. Clonality, and B. Clonality, which respectively gave AUC values above 0.5 in eight, nine, and seven immunotherapy cohorts. The predictive value of CD147 was, however, lower than the MSI score (AUC > 0.5 in 13 immunotherapy cohorts), CD274 (AUC > 0.5 in 21 immunotherapy cohorts), TIDE (AUC > 0.5 in 18 immunotherapy cohorts), IFNG (AUC > 0.5 in 17 immunotherapy cohorts), and CD8 (AUC > 0.5 in 18 immunotherapy cohorts). The correlation between CD147 levels and drug sensitivity based on the GDSC dataset indicated that methotrexate, TPCA-1 (IKK-2 inhibitor), and PAC-1 (first procaspase activating compound) were the top three drugs that positively related to CD147 expression (**Figure 9C** and **Table S2**; P<0.0001). On the contrary, bleomycin, 17-AAG, and docetaxel were the top three drugs that negatively related to CD147 expression (**Figure 9C** and **Supplementary Table 2**; P<0.0001). The correlation between CD147 levels and drug sensitivity based on the CTRP dataset showed that LRRK2-IN-1 (potent and selective inhibitor of LRRK2), PRIMA-1 (mutant p53 reactivator), and tacedinaline (inhibitor of HDAC) were the top three drugs which positively related to CD147 expression (**Figure 9D** and **Supplementary Table 3**; P<0.0001). These results demonstrated the immunotherapy value of CD147 in pan-cancer. More importantly, a series of targeted and small molecule drugs with promising therapeutic effects were predicted, which might provide a direction for immunotherapy targeting CD147 in pan-cancer.

## Discussion

CD147, as an important member of the immunoglobulin superfamily, participates in many physiological and pathological progress, including lymphocyte response, spermatogenesis, neurological development, cell proliferation, apoptosis, and cancer migration, metastasis, and differentiation (22–24). Accumulating evidence demonstrated that CD147 could promote tumor progression through regulating cancer cell apoptosis. Moreover, CD147 acts as a key role in mediating tumor cell invasiveness *via* regulating MMP expression, such as MMP-2 and MMP-9 in adjacent cancer cells or CAFs (25, 26). One study indicated that CD147 could induce malignant melanoma cell apoptosis through IGFBP2 and PTEN/PI3K/AKT signaling pathway (27). In a HNSCC xenograft model, Binbin Yu et al. concluded that overexpressed CD147 was associated with malignant clinicopathologic features (28). Meanwhile, CD147 was found to regulate tumor initiation and progression through nuclear factor kappa B (NF-κB) signaling. The underlying mechanism by which CD147 facilitates tumorigenesis, however, remains unclear. In this study, we explored the expression and mutant status of CD147 in pan-cancer. In most cancers, CD147 levels significantly increased in cancer samples than those of normal controls, which was closely related to the patients’ outcome. These results, combined with previous studies, emphasized the excellent predictive value of CD147 in a variety of cancers.

The immune system was first determined as a useful tool for targeting neoplastic disease by Wilhelm Bush and Friedrich Fehleisen in the nineteenth century (29). Since then, increasing studies have devoted to exploring the mechanism of tumor immunity for developing tumor immunotherapy (9, 30). Tumor immunotherapy, differing from previous standards of therapies (including surgery, chemotherapy, and radiotherapy), has brought patients significant improvements in terms of quality of life and survival outcome (31). Tumor immunotherapy has been recently categorized into immune checkpoint inhibitors, T-cell transfer therapy, monoclonal antibodies, therapeutic vaccines, and immune system modulators, among which immune checkpoint inhibitors have become the most dazzling star. Especially with the increasing popularity of high-throughput sequencing, several new immune checkpoints have been discovered (32–34). In this paper, we systematically explored the role of CD147 as a novel immunotherapy target in the TME among 33 cancer types. We found that CD147 was closely related to the immune score, estimate score, and stromal score in many cancers. In addition, the positive relationship between CD147 and neoantigens, MSI, TMB was revealed in these cancers. Moreover, the positive correlation between CD147 and other classic immune checkpoint molecules, such as SIGLEC15, TIGIT, CD274, HAVCR2, PDCD1, CTLA4, LAG3, and PDCD1LG2, was determined.

Increasing evidence has suggested that tumor-infiltrating immune cells in the TME play a critical role against cancer (35, 36). Cancer cells are able to escape from the immune response and build complicated microenvironment in which some immune cells may promote tumor progression, invasion, and resistance to therapy (34, 37). As the major component of the TME, tumor-associated macrophages (TAMs) are the main regulator in cancer-related inflammation (37, 38). Lots of basic and clinical researches have revealed that high infiltration of TAMs is associated with poor outcome (39). TAMs were found to participate in tumor immunity through regulating the functions of other immune cells and secreting cytokines that interact with immune checkpoints (40, 41). TAMs could polarize into two subtypes, M1 type and M2 type macrophages, which play opposite roles in tumor immunity. M1 subtype, known as pro-inflammatory macrophages, could eliminate tumor cells through direct or antibody-dependent cell-mediated cytotoxicity (42). M2 subtype, so-called anti-inflammatory macrophages, could facilitate survival and migration of tumor cell through expressing a variety of cytokines and growth factors, such as TGF-β1 (43).

T lymphocytes are the most effective mediators of the adaptive anti-tumor immune response. T lymphocytes mediate tumor immunity by secreting various cytokines into the TME, such as IL-2, IL-4, IL-5, IL17, IL-21, IL-22, and IFN-γ (44, 45). In particular, regulatory T (Treg) cells, known as the immunosuppressive class of CD4^+^ T cells, could suppress anti-cancer immunity by expressing CD25 and FOXP3 (46). NK cells not only identify and kill tumor cells by releasing cytolytic granules but also manage anti-tumor immune response by secreting specific chemokines (47). Recent findings found that B cells have both positive and negative effects on tumor immune response (48). Among the non-neoplastic cells, CAFs are essential for tumor mesenchyme in the TME (49), which CAFs not only provide mesenchymal support for tumor cells but also modulate tumorigenesis in a context-dependent manner (50). In this paper, we explored the expression landscape of CD147 in tumor cells and stromal cells in pan-cancer. CD147 was highly expressed on cancer cells and stromal cells, especially M2 macrophages, macrophages, T cells, B cells, and CAFs, in most cancers. Meanwhile, multiplex immunofluorescence staining was used to verify the expression of CD147 on macrophages and M2 macrophages in these cancers, which CD147 was mainly expressed on CD163^+^ M2 macrophages in BLCA, LSCC, CESC, PSCC, TGCT, and PRAD. In addition, CD147 was also found to be widely expressed on CD68^+^ macrophages in UTUC, THCA, BLCA, CESC, OPV, and OV. These results demonstrated the significant connection between CD147 and tumor cells, stromal cells in the TME.

The use of public databases and computational models to identify the optimal individualized therapeutic drugs has been increasingly popular (51, 52). In this paper, we calculated the biomarker relevance of CD147 in 25 immunotherapy cohorts to verify its predictive value. Meanwhile, we also predicted the sensitive drugs from two public databases based on CD147 expression. Interestingly, we found that CD147 alone had an AUC of more than 0.5 in 12 immunotherapy cohorts. CD147 exhibited a higher predictive value than TMB, T. Clonality, and B. Clonality in 8, 9, and 7 immunotherapy cohorts, indicating the predictive value of CD147 in immunotherapy. More importantly, a series of targeted small molecule drugs with promising therapeutic effects were predicted, providing theoretical basis for developing drugs targeting CD147.

## Conclusion

We comprehensively explored the prognostic value and immune aspects of CD147 in pan-cancer. However, lack of functional and mechanistic studies at the cellular and immunological levels was the major limitation of our study. Given that, more in-depth functional and mechanistic studies are needed. In sum, therapies targeting CD147 in the tumor microenvironment are promising in improving and prolonging the survival of cancer patients.

## Data Availability Statement

The original contributions presented in the study are included in the article/**Supplementary Material**. Further inquiries can be directed to the corresponding authors.

## Author Contributions

Writing -Original Draft, Methodology, Validation, Visualization: JW-Z and HZ. Data Curation, Validation: ZW, ZD, PL, JZ, ZL, and WZ-P. Investigation: JY, YP, NZ, and WW. Conceptualization, Methodology, Supervision, Project Administration and Funding Acquisition: HZ, SF, and QC. All authors contributed to the article and approved the submitted version.

## Funding

This study is supported by the National Nature Science Foundation of China (NO.82073893, 82102848, 81703622, 82060667, 81903725); the China Postdoctoral Science Foundation (NO. 2018M633002); the Natural Science Foundation of Hunan Province (NO. 2018JJ3838, 2020JJ8111); the Hunan Provincial Health and Health Committee Foundation of China (C2019186); Xiangya Hospital Central South University postdoctoral foundation.

## Conflict of Interest

The authors declare that the research was conducted in the absence of any commercial or financial relationships that could be construed as a potential conflict of interest.

## Publisher’s Note

All claims expressed in this article are solely those of the authors and do not necessarily represent those of their affiliated organizations, or those of the publisher, the editors and the reviewers. Any product that may be evaluated in this article, or claim that may be made by its manufacturer, is not guaranteed or endorsed by the publisher.

## References

[1] Siegel RL Miller KD Fuchs HE Jemal A. Cancer Statistics 2021. CA Cancer J Clin (2021) 71(1):7–33. doi: 10.3322/caac.21654 33433946

[2] Sung H Ferlay J Siegel RL Laversanne M Soerjomataram I Jemal A. Global Cancer Statistics 2020: GLOBOCAN Estimates of Incidence and Mortality Worldwide for 36 Cancers in 185 Countries. CA Cancer J Clin (2021) 71(3):209–49. doi: 10.3322/caac.21660 33538338

[3] Bray F Ferlay J Soerjomataram I Siegel RL Torre LA Jemal A. Global Cancer Statistics 2018: GLOBOCAN Estimates of Incidence and Mortality Worldwide for 36 Cancers in 185 Countries. CA Cancer J Clin (2018) 68(6):394–424. doi: 10.3322/caac.21492 30207593

[4] Rahib L Wehner MR Matrisian LM Nead KT. Estimated Projection of US Cancer Incidence and Death to 2040. JAMA Netw Open (2021) 4(4):e214708. doi: 10.1001/jamanetworkopen.2021.4708 33825840 PMC8027914

[5] Gu X Zheng R Xia C Zeng H Zhang S Zou X. Interactions Between Life Expectancy and the Incidence and Mortality Rates of Cancer in China: A Population-Based Cluster Analysis. Cancer Commun (Lond) (2018) 38(1):44. doi: 10.1186/s40880-018-0308-x 29970165 PMC6029078

[6] Sun YM Wang Y Sun XX Chen J Gong ZP Meng HY. Clinical Efficacy of Immune Checkpoint Inhibitors in Older Non-Small-Cell Lung Cancer Patients: A Meta-Analysis. Front Oncol (2020) 10:558454. doi: 10.3389/fonc.2020.558454 33072584 PMC7538790

[7] Baghban R Roshangar L Jahanban-Esfahlan R Seidi K Ebrahimi-Kalan A Jaymand M. Tumor Microenvironment Complexity and Therapeutic Implications at a Glance. Cell Commun Signal (2020) 18(1):59. doi: 10.1186/s12964-020-0530-4 32264958 PMC7140346

[8] Taki M Abiko K Ukita M Murakami R Yamanoi K Yamaguchi K. Tumor Immune Microenvironment During Epithelial-Mesenchymal Transition. Clin Cancer Res (2021) 27(17):4669–79. doi: 10.1158/1078-0432.CCR-20-4459 33827891

[9] Zhang H Dai Z Wu W Wang Z Zhang N Zhang L. Regulatory Mechanisms of Immune Checkpoints PD-L1 and CTLA-4 in Cancer. J Exp Clin Cancer Res (2021) 40(1):184. doi: 10.1186/s13046-021-01987-7 34088360 PMC8178863

[10] Brunner-Weinzierl MC Rudd CE. CTLA-4 and PD-1 Control of T-Cell Motility and Migration: Implications for Tumor Immunotherapy. Front Immunol (2018) 9:2737. doi: 10.3389/fimmu.2018.02737 30542345 PMC6277866

[11] Kamada T Togashi Y Tay C Ha D Sasaki A Nakamura Y. PD-1(+) Regulatory T Cells Amplified by PD-1 Blockade Promote Hyperprogression of Cancer. Proc Natl Acad Sci USA (2019) 116(20):9999–10008. doi: 10.1073/pnas.1822001116 31028147 PMC6525547

[12] Xiong L Edwards CK 3rd Zhou L. The Biological Function and Clinical Utilization of CD147 in Human Diseases: A Review of the Current Scientific Literature. Int J Mol Sci (2014) 15(10):17411–41. doi: 10.3390/ijms151017411 PMC4227170 25268615

[13] Guo N Ye S Zhang K Yu X Cui H Yang X. A Critical Epitope in CD147 Facilitates Memory CD4(+) T-Cell Hyper-Activation in Rheumatoid Arthritis. Cell Mol Immunol (2019) 16(6):568–79. doi: 10.1038/s41423-018-0012-4 PMC6804595 29563614

[14] Gabison EE Huet E Baudouin C Menashi S. Direct Epithelial-Stromal Interaction in Corneal Wound Healing: Role of EMMPRIN/CD147 in MMPs Induction and Beyond. Prog Retin Eye Res (2009) 28(1):19–33. doi: 10.1016/j.preteyeres.2008.11.001 19056510

[15] Landras A Reger de Moura C Jouenne F Lebbe C Menashi S Mourah S. CD147 Is a Promising Target of Tumor Progression and a Prognostic Biomarker. Cancers (Basel) (2019) 11(11):1803. doi: 10.3390/cancers11111803 PMC6896083 31744072

[16] Kong LM Liao CG Zhang Y Xu J Li Y Huang W. A Regulatory Loop Involving miR-22, Sp1, and C-Myc Modulates CD147 Expression in Breast Cancer Invasion and Metastasis. Cancer Res (2014) 74(14):3764–78. doi: 10.1158/0008-5472.CAN-13-3555 24906624

[17] Wang C Xu C Niu R Hu G Gu Z Zhuang Z. MiR-890 Inhibits Proliferation and Invasion and Induces Apoptosis in Triple-Negative Breast Cancer Cells by Targeting CD147. BMC Cancer (2019) 19(1):577. doi: 10.1186/s12885-019-5796-9 31196010 PMC6567604

[18] Meng Y Fan XY Yang LJ Xu BQ He D Xu Z. Detachment Activated CyPA/CD147 Induces Cancer Stem Cell Potential in Non-Stem Breast Cancer Cells. Front Cell Dev Biol (2020) 8:543856. doi: 10.3389/fcell.2020.543856 33195186 PMC7640948

[19] Butler A Hoffman P Smibert P Papalexi E Satija R. Integrating Single-Cell Transcriptomic Data Across Different Conditions, Technologies, and Species. Nat Biotechnol (2018) 36(5):411–20. doi: 10.1038/nbt.4096 PMC6700744 29608179

[20] Zhang J Novakovic N Hua Y Keep RF Xi G. Role of Lipocalin-2 in Extracellular Peroxiredoxin 2-Induced Brain Swelling, Inflammation and Neuronal Death. Exp Neurol (2021) 335:113521. doi: 10.1016/j.expneurol.2020.113521 33129840 PMC7750274

[21] Zhou J. Pei X. Yang Y. Wang Z. Gao W. Ye R.. Orphan Nuclear Receptor TLX Promotes Immunosuppression via Its Transcriptional Activation of PD-L1 in Glioma. J Immunother Cancer (2021) 9(4). doi: 10.1136/jitc-2020-001937 PMC8055120 33858847

[22] Wang K Chen W Zhang Z Deng Y Lian JQ Du P. CD147-Spike Protein is a Novel Route for SARS-CoV-2 Infection to Host Cells. Signal Transduct Target Ther (2020) 5(1):283. doi: 10.1038/s41392-020-00426-x 33277466 PMC7714896

[23] Zhang T Li H Wang K Xu B Chen ZN Bian H. Deficiency of CD147 Attenuated Non-Alcoholic Steatohepatitis Progression in an NLRP3-Dependent Manner. Front Cell Dev Biol (2020) 8:784. doi: 10.3389/fcell.2020.00784 32903542 PMC7438480

[24] Pata S Surinkaew S Takheaw N Laopajon W Chuensirikulchai K Kasinrerk W. Differential CD147 Functional Epitopes on Distinct Leukocyte Subsets. Front Immunol (2021) 12:704309. doi: 10.3389/fimmu.2021.704309 34421910 PMC8371324

[25] Cui J Huang W Wu B Jin J Jing L Shi WP. N-Glycosylation by N-Acetylglucosaminyltransferase V Enhances the Interaction of CD147/basigin With Integrin Beta1 and Promotes HCC Metastasis. J Pathol (2018) 245(1):41–52. doi: 10.1002/path.5054 29431199 PMC5947728

[26] Liu N Qi M Li K Zeng W Li J Yin M. CD147 Regulates Melanoma Metastasis *via* the NFAT1-MMP-9 Pathway. Pigment Cell Melanoma Res (2020) 33(5):731–43. doi: 10.1111/pcmr.12886 32339381

[27] Zhao S Wu L Kuang Y Su J Luo Z Wang Y. Downregulation of CD147 Induces Malignant Melanoma Cell Apoptosis *via* the Regulation of IGFBP2 Expression. Int J Oncol (2018) 53(6):2397–408. doi: 10.3892/ijo.2018.4579 PMC6203154 30272281

[28] Yu B Zhang Y Wu K Wang L Jiang Y Chen W. CD147 Promotes Progression of Head and Neck Squamous Cell Carcinoma *via* NF-Kappa B Signaling. J Cell Mol Med (2019) 23(2):954–66. doi: 10.1111/jcmm.13996 PMC6349162 30421493

[29] Dobosz P Dzieciatkowski T. The Intriguing History of Cancer Immunotherapy. Front Immunol (2019) 10:2965. doi: 10.3389/fimmu.2019.02965 31921205 PMC6928196

[30] Yang K Wu Z Zhang H Zhang N Wu W Wang Z. Glioma Targeted Therapy: Insight Into Future of Molecular Approaches. Mol Cancer (2022) 21(1):39. doi: 10.1186/s12943-022-01513-z 35135556 PMC8822752

[31] Esfahani K Roudaia L Buhlaiga N Del Rincon SV Papneja N Miller WH Jr. A Review of Cancer Immunotherapy: From the Past, to the Present, to the Future. Curr Oncol (2020) 27(Suppl 2):S87–97. doi: 10.3747/co.27.5223 PMC7194005 32368178

[32] Liang X Wang Z Dai Z Zhang H Cheng Q Liu Z. Promoting Prognostic Model Application: A Review Based on Gliomas. J Oncol (2021) 2021:7840007. doi: 10.1155/2021/7840007 34394352 PMC8356003

[33] Wang Z Liu Y Mo Y Zhang H Dai Z Zhang X. The CXCL Family Contributes to Immunosuppressive Microenvironment in Gliomas and Assists in Gliomas Chemotherapy. Front Immunol (2021) 12:731751. doi: 10.3389/fimmu.2021.731751 34603309 PMC8482424

[34] Zhang H Wang Z Dai Z Wu W Cao H Li S. Novel Immune Infiltrating Cell Signature Based on Cell Pair Algorithm Is a Prognostic Marker in Cancer. Front Immunol (2021) 12:694490. doi: 10.3389/fimmu.2021.694490 34594324 PMC8476752

[35] Galli F Aguilera JV Palermo B Markovic SN Nistico P Signore A. Relevance of Immune Cell and Tumor Microenvironment Imaging in the New Era of Immunotherapy. J Exp Clin Cancer Res (2020) 39(1):89. doi: 10.1186/s13046-020-01586-y 32423420 PMC7236372

[36] Hiam-Galvez KJ Allen BM Spitzer MH. Systemic Immunity in Cancer. Nat Rev Cancer (2021) 21(6):345–59. doi: 10.1038/s41568-021-00347-z PMC8034277 33837297

[37] Zhang H Luo YB Wu W Zhang L Wang Z Dai Z. The Molecular Feature of Macrophages in Tumor Immune Microenvironment of Glioma Patients. Comput Struct Biotechnol J (2021) 19:4603–18. doi: 10.1016/j.csbj.2021.08.019 PMC8383063 34471502

[38] Chen Y Song Y Du W Gong L Chang H Zou Z. Tumor-Associated Macrophages: An Accomplice in Solid Tumor Progression. J BioMed Sci (2019) 26(1):78. doi: 10.1186/s12929-019-0568-z 31629410 PMC6800990

[39] Mantovani A Marchesi F Malesci A Laghi L Allavena P. Tumour-Associated Macrophages as Treatment Targets in Oncology. Nat Rev Clin Oncol (2017) 14(7):399–416. doi: 10.1038/nrclinonc.2016.217 28117416 PMC5480600

[40] Murray PJ Allen JE Biswas SK Fisher EA Gilroy DW Goerdt S. Macrophage Activation and Polarization: Nomenclature and Experimental Guidelines. Immunity (2014) 41(1):14–20. doi: 10.1016/j.immuni.2014.06.008 25035950 PMC4123412

[41] Sun X Ingman WV. Cytokine Networks That Mediate Epithelial Cell-Macrophage Crosstalk in the Mammary Gland: Implications for Development and Cancer. J Mammary Gland Biol Neoplasia (2014) 19(2):191–201. doi: 10.1007/s10911-014-9319-7 24924120

[42] Pan Y Yu Y Wang X Zhang T. Tumor-Associated Macrophages in Tumor Immunity. Front Immunol (2020) 11:583084. doi: 10.3389/fimmu.2020.583084 33365025 PMC7751482

[43] Yin M Li X Tan S Zhou HJ Ji W Bellone S. Tumor-Associated Macrophages Drive Spheroid Formation During Early Transcoelomic Metastasis of Ovarian Cancer. J Clin Invest (2016) 126(11):4157–73. doi: 10.1172/JCI87252 PMC5096908 27721235

[44] Walsh SR Simovic B Chen L Bastin D Nguyen A Stephenson K. Endogenous T Cells Prevent Tumor Immune Escape Following Adoptive T Cell Therapy. J Clin Invest (2019) 129(12):5400–10. doi: 10.1172/JCI126199 PMC6877330 31682239

[45] St Paul M Ohashi PS. The Roles of CD8(+) T Cell Subsets in Antitumor Immunity. Trends Cell Biol (2020) 30(9):695–704. doi: 10.1016/j.tcb.2020.06.003 32624246

[46] Josefowicz SZ Lu LF Rudensky AY. Regulatory T Cells: Mechanisms of Differentiation and Function. Annu Rev Immunol (2012) 30:531–64. doi: 10.1146/annurev.immunol.25.022106.141623 PMC6066374 22224781

[47] Shimasaki N Jain A Campana D. NK Cells for Cancer Immunotherapy. Nat Rev Drug Discovery (2020) 19(3):200–18. doi: 10.1038/s41573-019-0052-1 31907401

[48] Largeot A Pagano G Gonder S Moussay E Paggetti J. The B-Side of Cancer Immunity: The Underrated Tune. Cells (2019) 8(5):449. doi: 10.3390/cells8050449 PMC6562515 31086070

[49] Biffi G Tuveson DA. Deciphering Cancer Fibroblasts. J Exp Med (2018) 215(12):2967–8. doi: 10.1084/jem.20182069 PMC6279394 30470718

[50] Liu T Han C Wang S Fang P Ma Z Xu L. Cancer-Associated Fibroblasts: An Emerging Target of Anti-Cancer Immunotherapy. J Hematol Oncol (2019) 12(1):86. doi: 10.1186/s13045-019-0770-1 31462327 PMC6714445

[51] Adam G Rampasek L Safikhani Z Smirnov P Haibe-Kains B Goldenberg A. Machine Learning Approaches to Drug Response Prediction: Challenges and Recent Progress. NPJ Precis Oncol (2020) 4:19. doi: 10.1038/s41698-020-0122-1 32566759 PMC7296033

[52] Rydzewski NR Peterson E Lang JM Yu M Laura Chang S Sjostrom M. Predicting Cancer Drug TARGETS - TreAtment Response Generalized Elastic-neT Signatures. NPJ Genom Med (2021) 6(1):76. doi: 10.1038/s41525-021-00239-z 34548481 PMC8455625

