# Supplementary material for: Large-Scale Single-Cell and Bulk Sequencing Analyses Reveal the Prognostic Value and Immune Aspects of CD147 in Pan-Cancer
Source: Front Immunol. 2022 Apr 6;13:810471. doi: 10.3389/fimmu.2022.810471 (PMC9019465; doi:10.3389/fimmu.2022.810471)
Supplement: Supplementary file 1 [file DataSheet_1.docx]

**[Supplementary](https://www.frontiersin.org/articles/10.3389/fimmu.2021.643282/full#h15) Information**

**
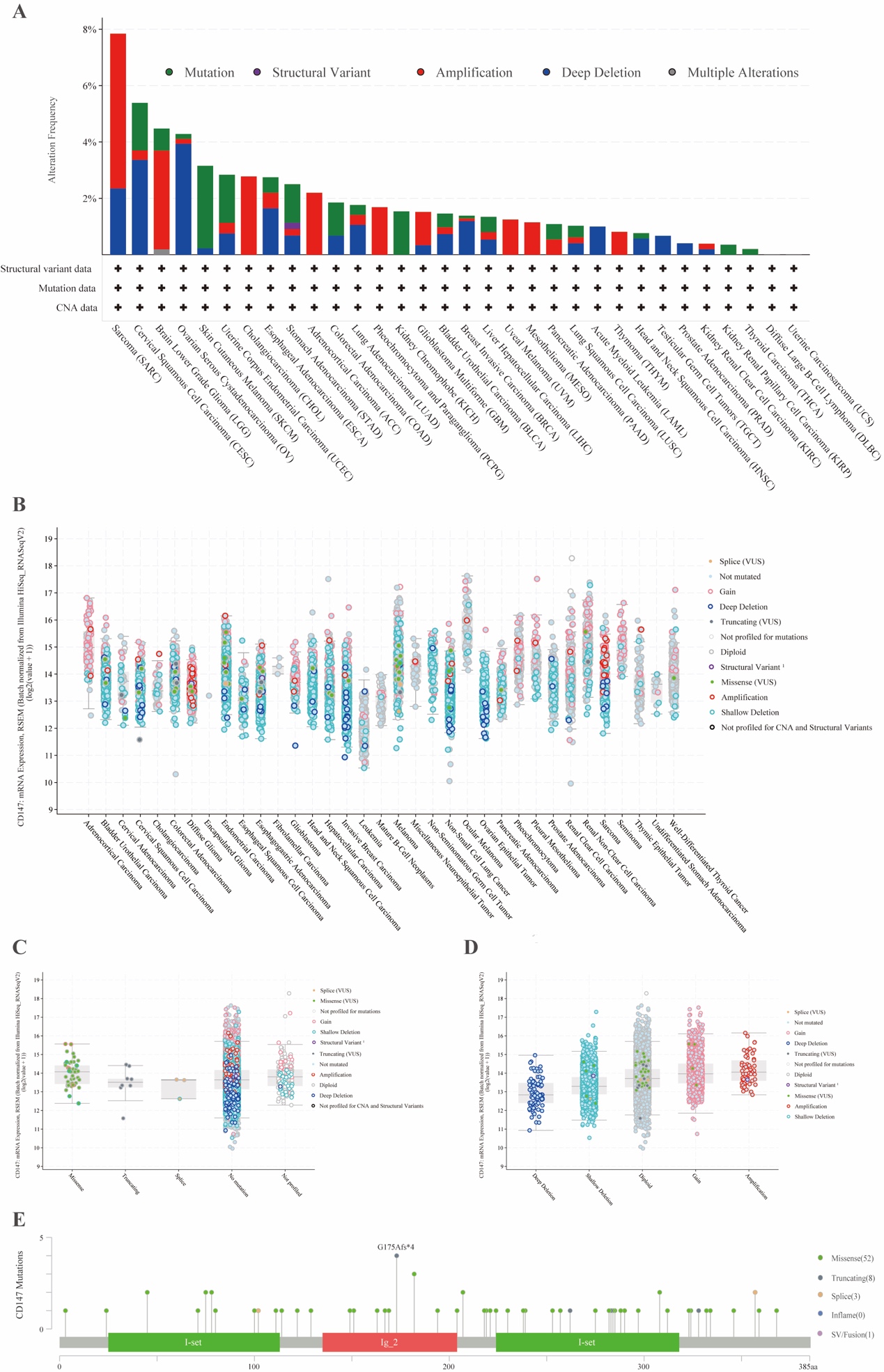
**

[**Supplementary**](https://www.frontiersin.org/articles/10.3389/fimmu.2021.643282/full#h15) **Figure 1. Mutation aspect of CD147 in pan-cancer. Mutation frequency of CD147 in pan-cancer. (A). The entire mutation count of CD147 from the TCGA database based on the cBioPortal analysis (B). The relationship between CD147 mRNA levels and mutant types (C) and copy-number alterations (D). Mutation profile of CD147 across protein domains (E). ACC: Adrenocortical carcinoma; BRCA: Breast invasive carcinoma; CESC: Cervical squamous cell carcinoma and endocervical adenocarcinoma; CHOL: Cholangiocarcinoma; COAD: Colon adenocarcinoma; ESCA: Esophageal carcinoma; GBM: Glioblastoma; HNSCC: Head and neck squamous cell carcinomas; KICH: Kidney chromophobe; KIRC: Kidney renal clear cell carcinoma; KIRP: Kidney renal papillary cell carcinoma; LAML: Acute myeloid leukemia; LGG: Low grade glioma; LIHC: Liver hepatocellular carcinoma; LUSC: Lung squamous cell carcinoma; OV: Ovarian Cancer; PAAD: Pancreatic adenocarcinoma; PCPG: Pheochromocytoma, and paraganglioma; PRAD: Prostate adenocarcinoma; READ: Rectum adenocarcinoma; SARC: Sarcoma; SKCM: Skin cutaneous melanoma; STAD: Stomach adenocarcinoma; TGCT: Testicular germ cell tumors; THCA: Thyroid carcinoma; THYM: Thymoma; ; UCS: Uterine carcinosarcoma; UVM: Uveal melanoma.**

**
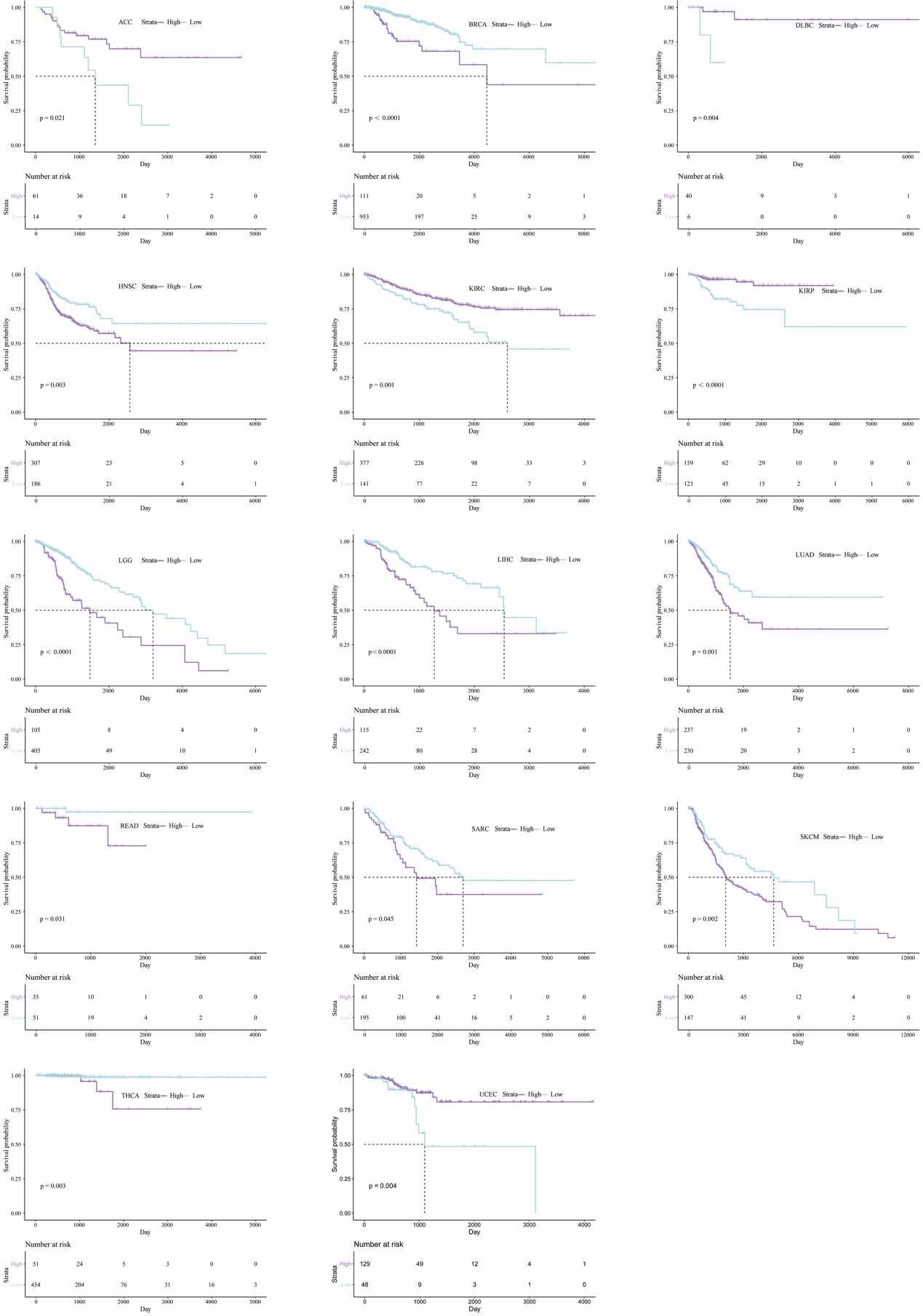
**

[**Supplementary**](https://www.frontiersin.org/articles/10.3389/fimmu.2021.643282/full#h15) **Figure 2. KM displayed the prognostic value of CD147 on DSS in pan-cancer.**

**
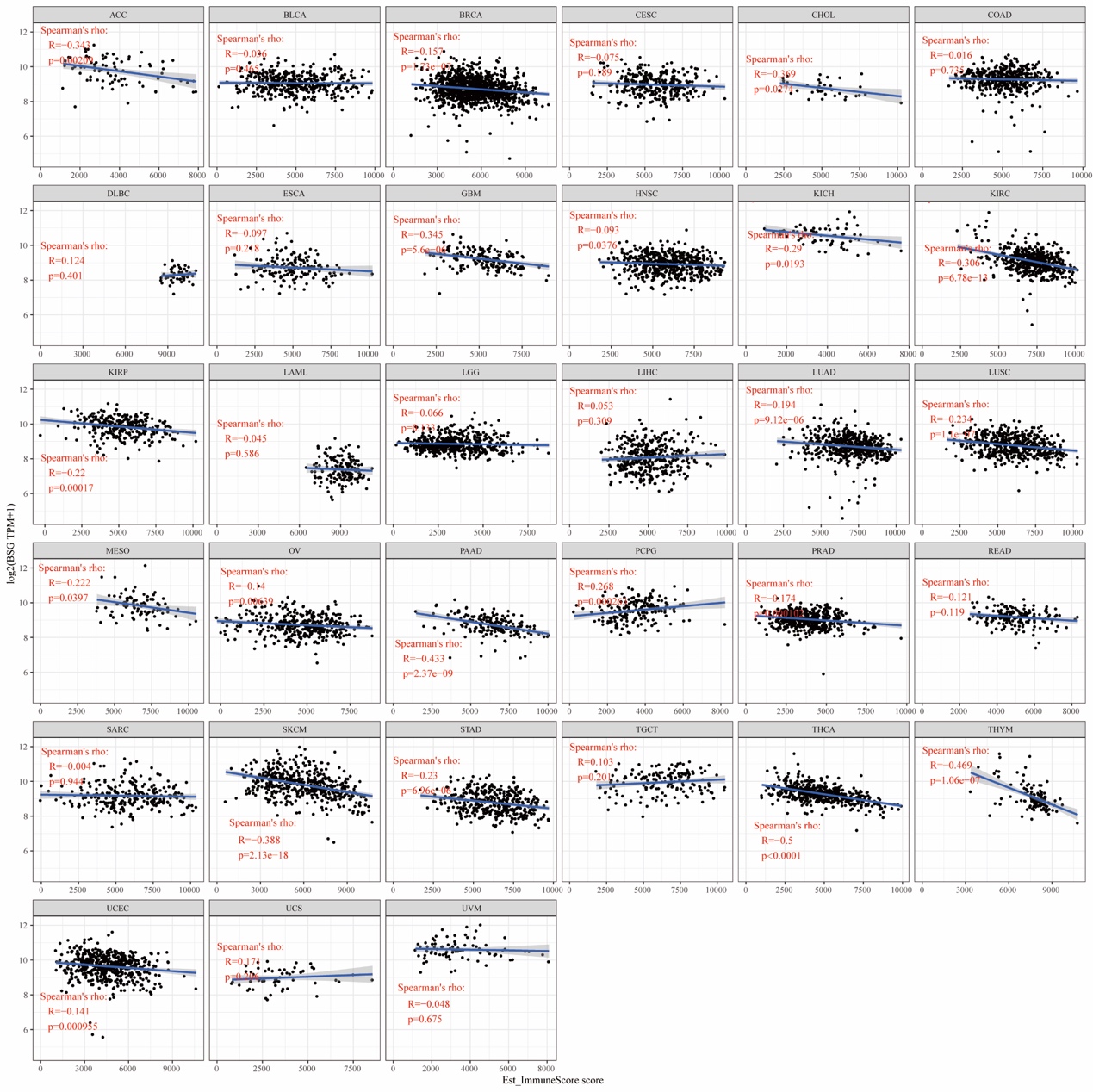
**

[**Supplementary**](https://www.frontiersin.org/articles/10.3389/fimmu.2021.643282/full#h15) **Figure 3. Relationship between CD147 expression and the immune scores.**

**
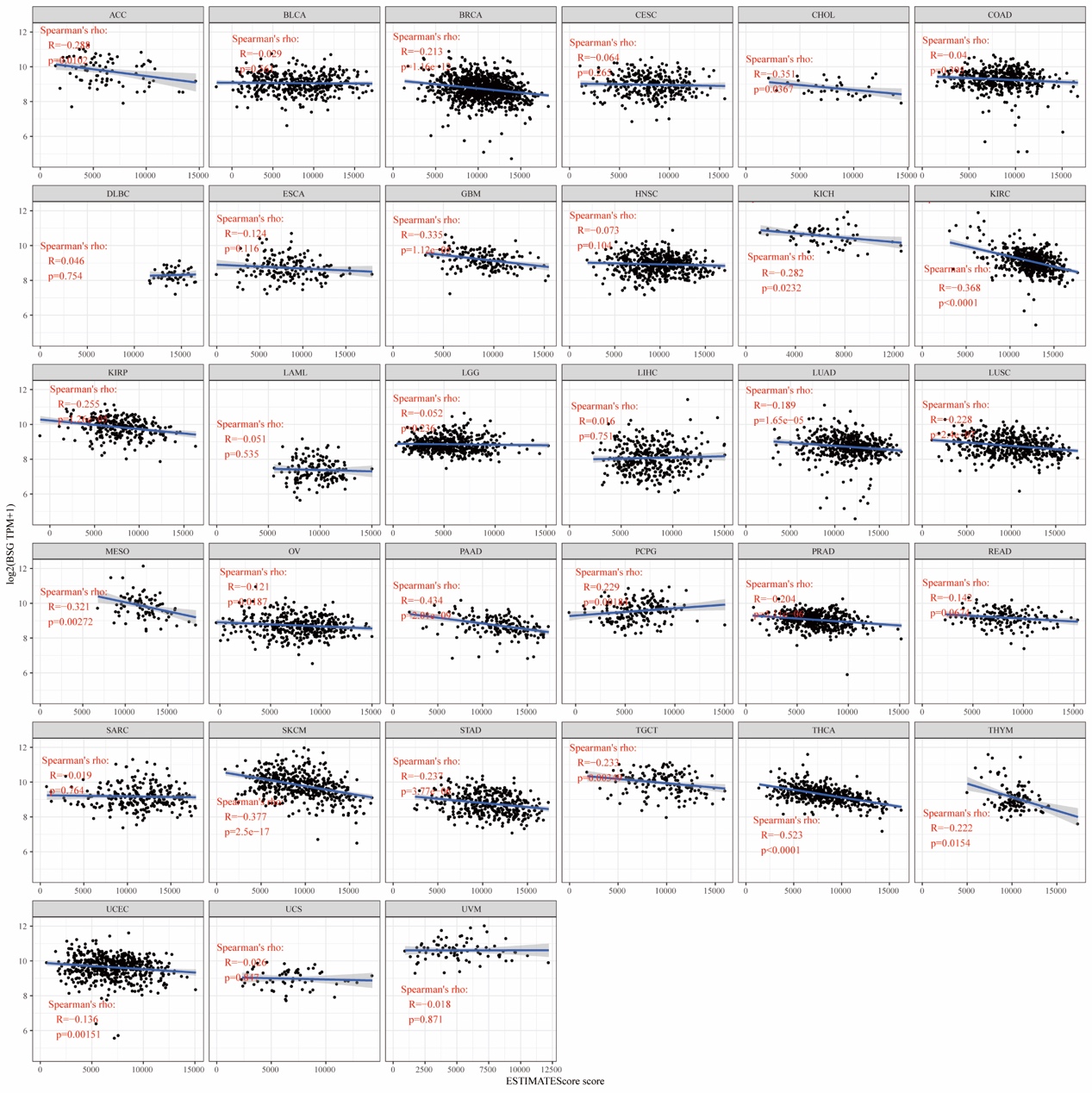
**

[**Supplementary**](https://www.frontiersin.org/articles/10.3389/fimmu.2021.643282/full#h15) **Figure 4. Relationship between CD147 expression and the estimate scores.**

**
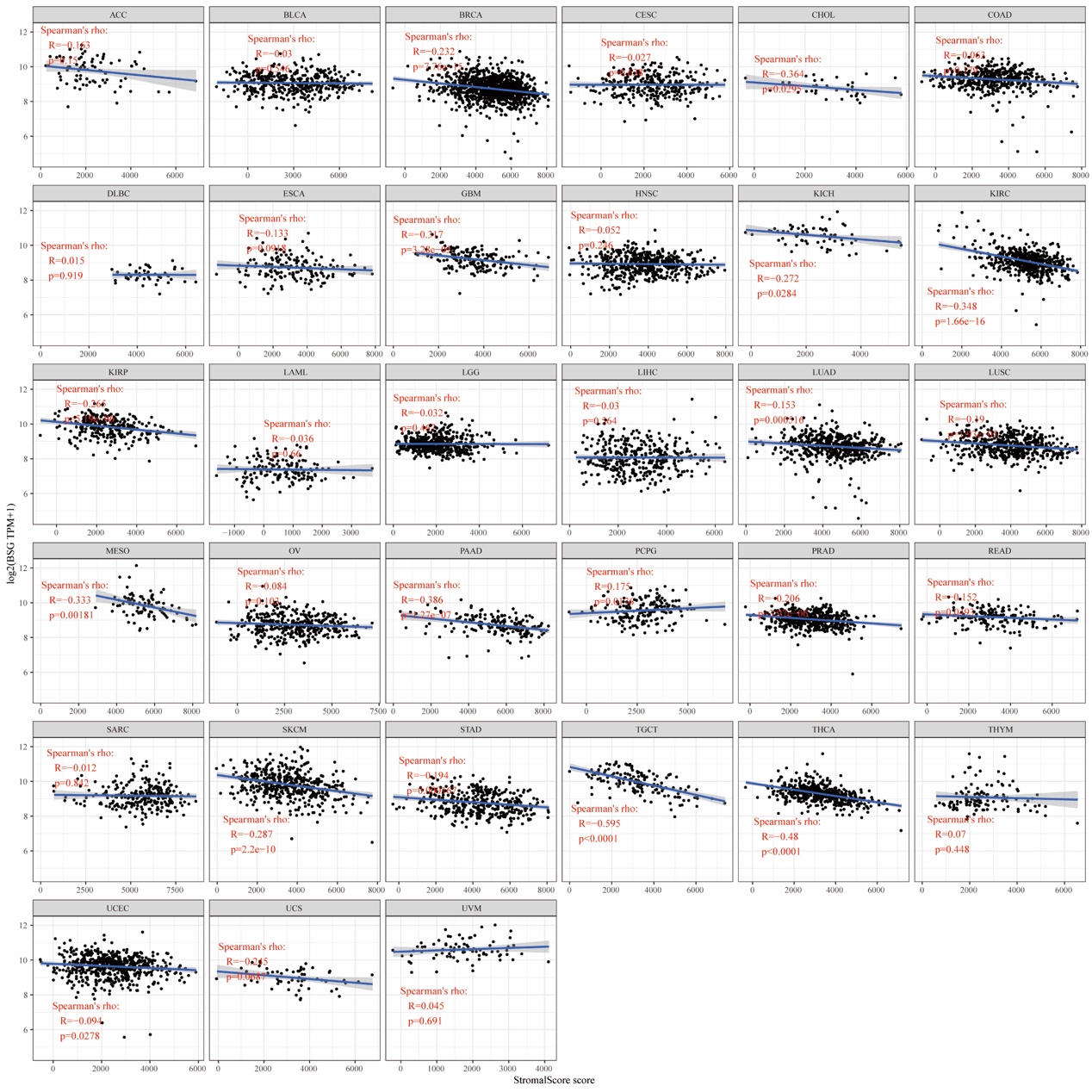
**

[**Supplementary**](https://www.frontiersin.org/articles/10.3389/fimmu.2021.643282/full#h15) **Figure 5. Relationship between CD147 expression and the stromal scores.**

**
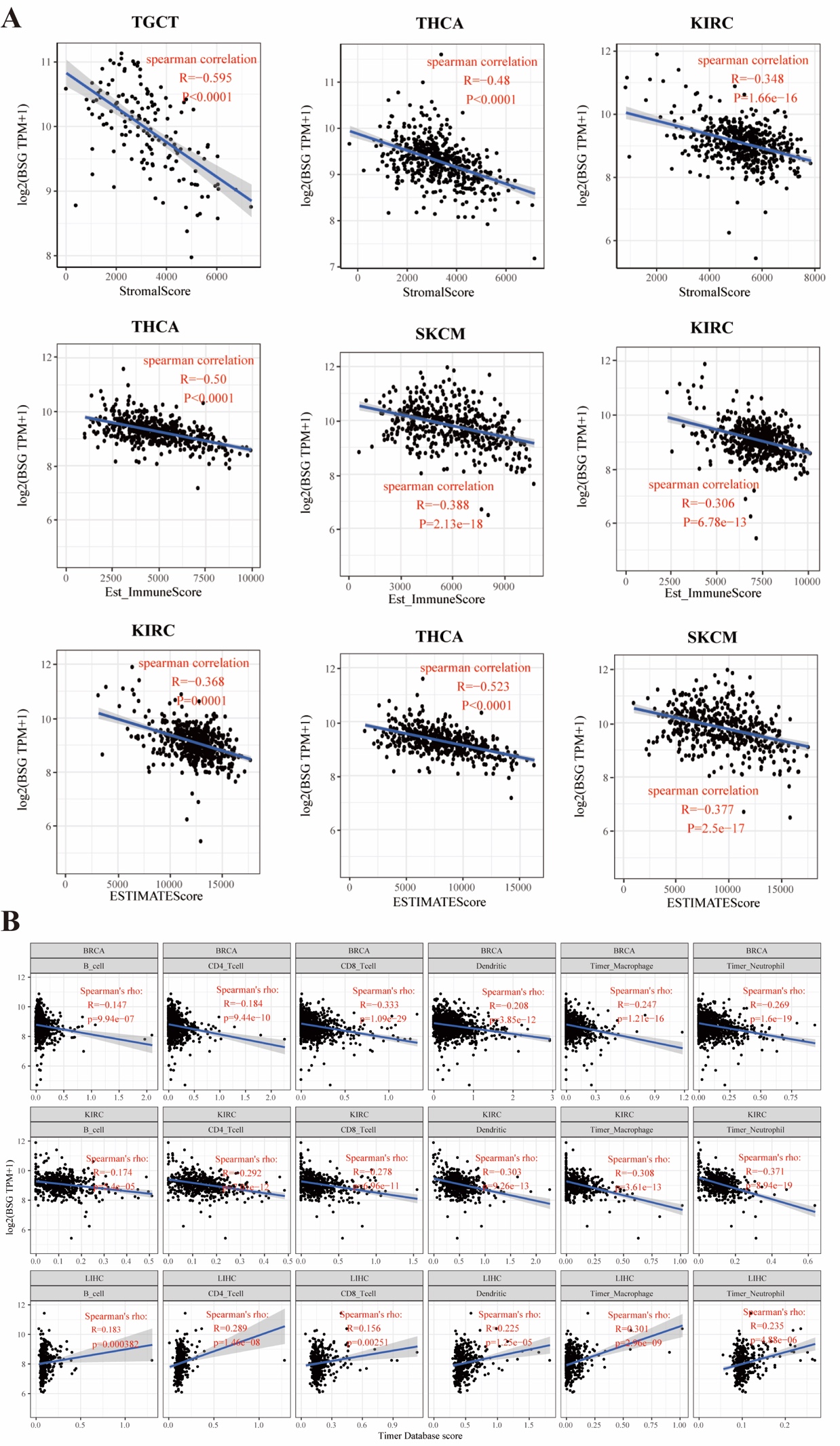
**

[**Supplementary**](https://www.frontiersin.org/articles/10.3389/fimmu.2021.643282/full#h15) **Figure 6. Correlation of CD147 expression with immune infiltrates in TME. Top three cancers that positively correlated with stromal score, immune score and estimate score based on CD147 levels (A). Top three cancers related to the infiltrating immune cells in the TME based on CD147 levels (B).**

**
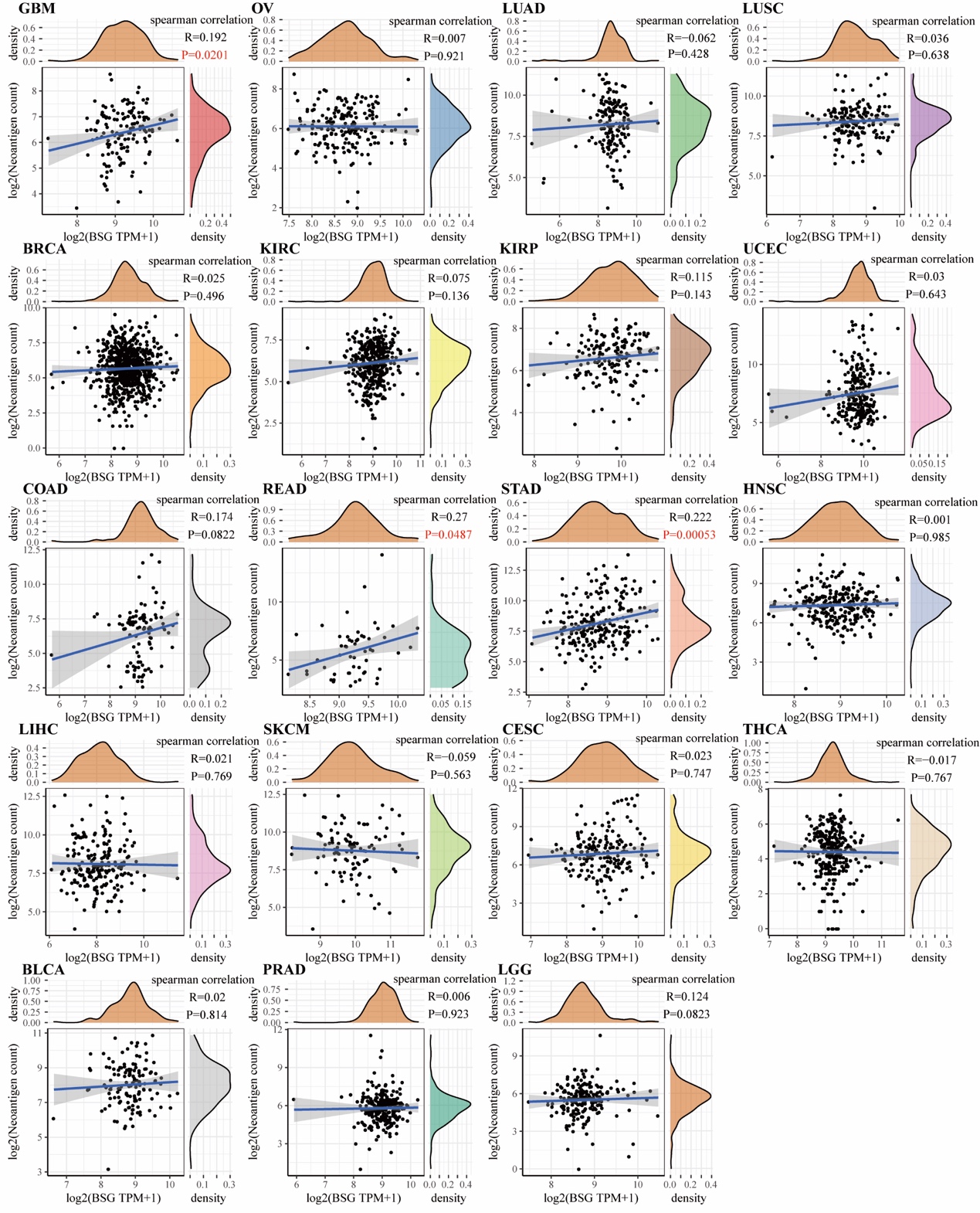
**

[**Supplementary**](https://www.frontiersin.org/articles/10.3389/fimmu.2021.643282/full#h15) **Figure 7. Relationship between CD147 levels and neoantigens in pan-cancer.**

**
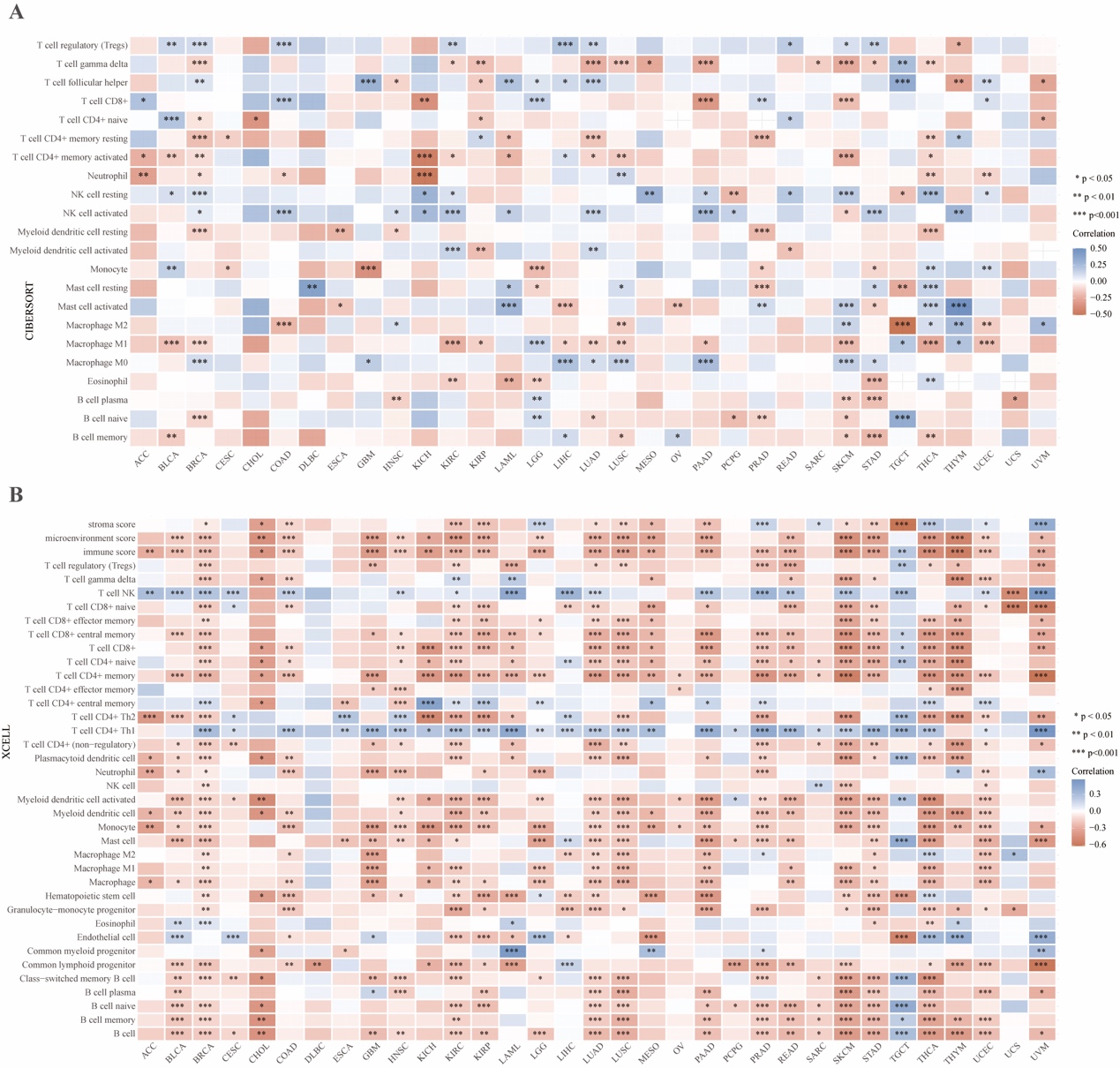
**

[**Supplementary**](https://www.frontiersin.org/articles/10.3389/fimmu.2021.643282/full#h15) **Figure 8. Relationship between CD147 levels and immune infiltrates analyzed by the R package immunedeconv in the TME. Immune cell infiltration analyzed by the CIBERSORT (A) and xCell (B) algorithms. *p< 0.05, **p < 0.01, ***p < 0.001.**

**
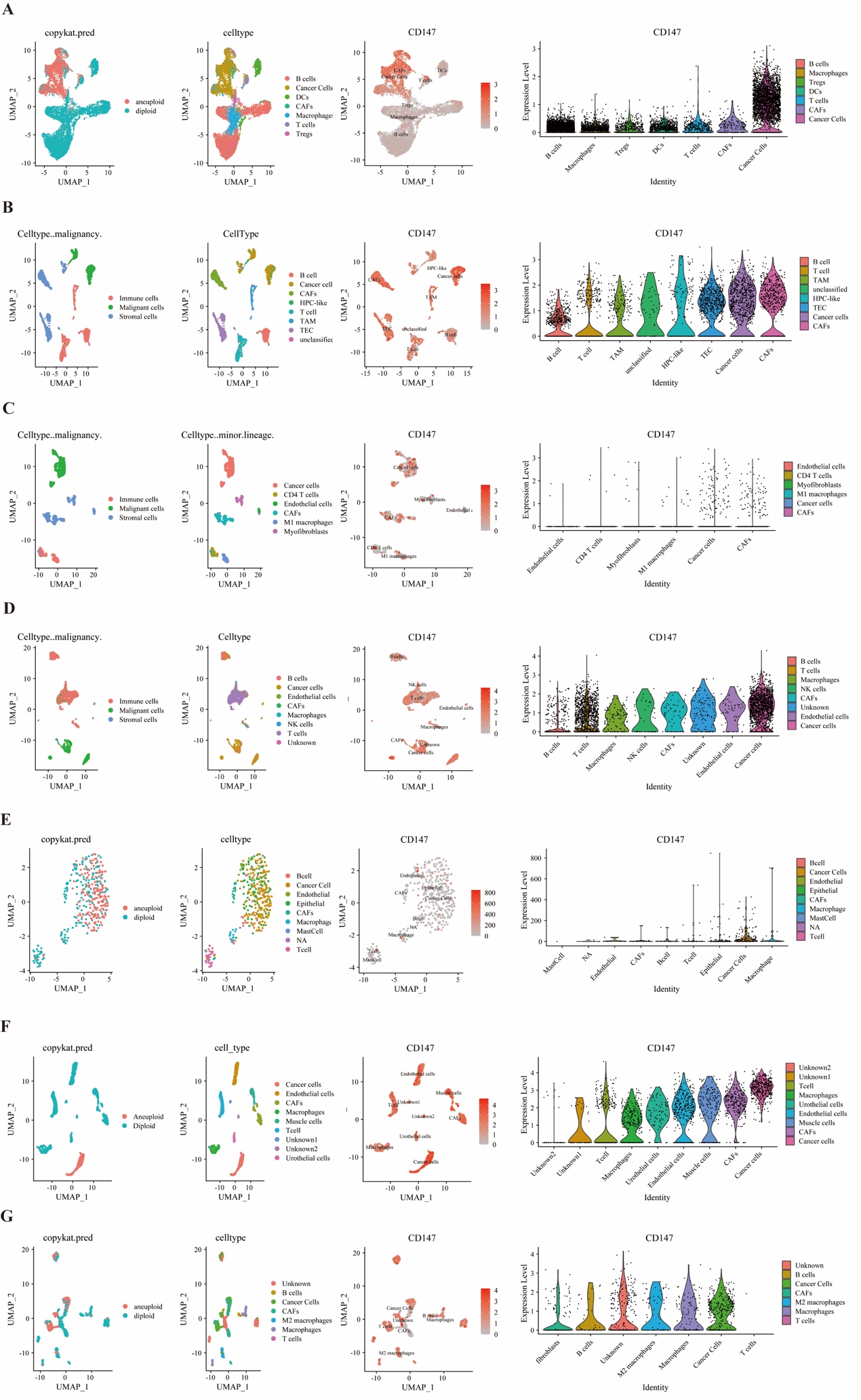
**

[**Supplementary**](https://www.frontiersin.org/articles/10.3389/fimmu.2021.643282/full#h15) **Figure 9. Single cell sequencing analyzing CD147 co-expression on tumor and stromal cells in pan-cancer. The expression levels of CD147 in tumor and stromal cells in STAD (A), LIHC (B), OV (C), SKCM (D), COAD (E), BLCA (F), and BRCA (G).**

**
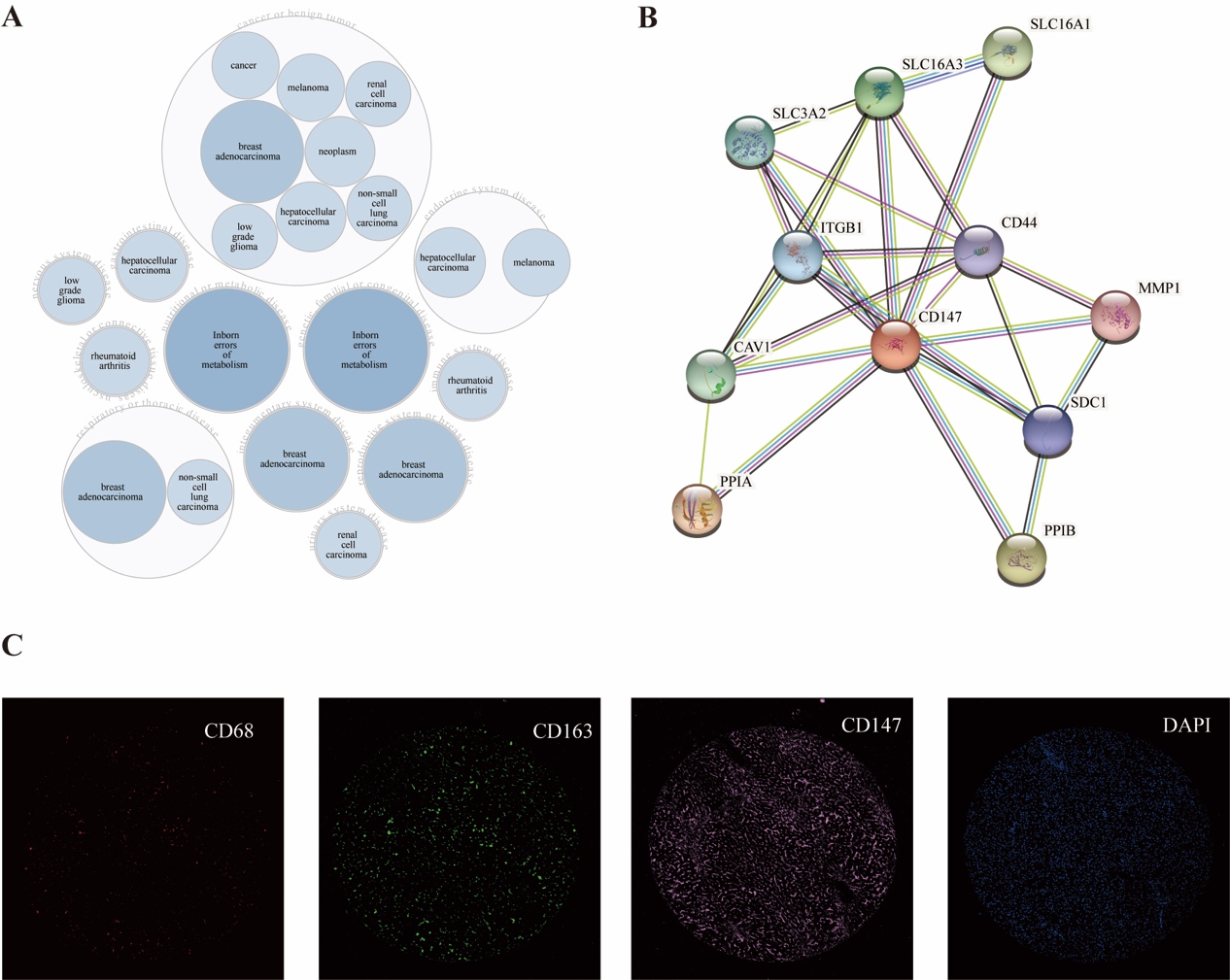
**

[**Supplementary**](https://www.frontiersin.org/articles/10.3389/fimmu.2021.643282/full#h15) **Figure 10. The involvement of CD147 in diseases based on the OPENTARGET platform (A). The PPI network of CD147 with other molecules (B). Markers of macrophage, M2 macrophage, CD147, and DAPI. CD68 was marked red, CD163 was marked green, CD147 was marked purple, and DAPI was marked blue (C).**

[**Supplementary**](https://www.frontiersin.org/articles/10.3389/fimmu.2021.643282/full#h15) **Table 1. The list of the sample ids or dataset ids in this study.**

[**Supplementary**](https://www.frontiersin.org/articles/10.3389/fimmu.2021.643282/full#h15) **Table 2. The correlation between CD147 expression and the sensitivity of GDSC drugs (top 30) in pan-cancer.**

[**Supplementary**](https://www.frontiersin.org/articles/10.3389/fimmu.2021.643282/full#h15) **Table 3. The correlation between CD147 expression and the sensitivity of CTRP drugs (top 30) in pan-cancer.**
